# Comparison of variance estimators for meta-analysis of instrumental variable estimates

**DOI:** 10.1093/ije/dyw123

**Published:** 2016-09-02

**Authors:** AF Schmidt, AD Hingorani, BJ Jefferis, J White, RHH Groenwold, F Dudbridge, for the UCLEB Consortium

**Affiliations:** 1 Institute of Cardiovascular Science; 2 Department of Primary Care and Population Health; 3 UCL Genetics Institute, University College London, London, UK; 4 Julius Center for Health Sciences and Primary Care, University Medical Center Utrecht, Utrecht, The Netherlands; 5 Department of Non-Communicable Disease Epidemiology, London School of Hygiene and Tropical Medicine, London, UK

**Keywords:** Epidemiology methods, Mendelian randomization analysis, statistics

## Abstract

**Background:** Mendelian randomization studies perform instrumental variable (IV) analysis using genetic IVs. Results of individual Mendelian randomization studies can be pooled through meta-analysis. We explored how different variance estimators influence the meta-analysed IV estimate.

**Methods:** Two versions of the delta method (IV before or after pooling), four bootstrap estimators, a jack-knife estimator and a heteroscedasticity-consistent (HC) variance estimator were compared using simulation. Two types of meta-analyses were compared, a two-stage meta-analysis pooling results, and a one-stage meta-analysis pooling datasets.

**Results:** Using a two-stage meta-analysis, coverage of the point estimate using bootstrapped estimators deviated from nominal levels at weak instrument settings and/or outcome probabilities ≤ 0.10. The jack-knife estimator was the least biased resampling method, the HC estimator often failed at outcome probabilities ≤ 0.50 and overall the delta method estimators were the least biased. In the presence of between-study heterogeneity, the delta method before meta-analysis performed best. Using a one-stage meta-analysis all methods performed equally well and better than two-stage meta-analysis of greater or equal size.

**Conclusions:** In the presence of between-study heterogeneity, two-stage meta-analyses should preferentially use the delta method before meta-analysis. Weak instrument bias can be reduced by performing a one-stage meta-analysis.

## Introduction

Despite considerable effort, observational (i.e. nonrandomized) studies are sensitive to confounding bias and reverse causation.^1^^-^^4^ To overcome these problems, Mendelian randomization (MR) studies have been advocated, using one or multiple single nucleotide polymorphisms (SNPs) as an instrument in instrumental variable (IV) analyses.^5^^,^^6^

In this type of Mendelian randomization study, the effects of an IV on an intermediate phenotype and on an outcome are estimated and combined to derive the causal effect of the intermediate on the outcome. This causal effect is unbiased if (among others) the following three assumptions hold: (i) the IV is associated with phenotype; (ii) conditional on the phenotype and the (possibly unmeasured) confounders, the IV is independent of the outcome; and (iii) the IV is independent of confounders.^7^

Although the performance of the different IV point estimators has previously been explored,^8^^,^^9^ the performance of the different variance estimators remains unclear. This is especially important because, to increase precision, Mendelian randomization studies often meta-analyse results from multiple studies. Because of this, different variance estimators not only impact type-1 error rates and confidence intervals but may also lead to different point estimates.

Typically, three types of meta-analysis can be defined: an aggregated meta-analysis combining study specific results; a two-stage individual patient data meta-analysis, in which an analysis script is designed and shared prospectively, before pooling study specific results; an one-stage individual patient data meta-analysis sharing the actual datasets. Given the usually straightforward analyses in genetic epidemiology, the differences between aggregated meta-analysis and two-stage individual patient data meta-analysis are often small; therefore here we only differentiate between two-stage meta-analyses and one-stage meta-analyses. A recent review by Boef *et al*.^10^ showed that 47 out of 80 meta-analyses of Mendelian randomization performed a two-staged analysis; among those, 10 performed IV analysis within each study before combining, whereas 9 combined gene-phenotype and gene-outcome associations separately before performing IV analysis. We note that gene scores are also used as instruments;^11^ using aggregated results this can be implemented, for example, by meta-analysing aggregated results of the gene-biomarker and the gene-outcome relationships into two estimates^12^ and applying the ratio estimator (see Methods). Alternatively, when individual patient data are available, gene scores can be implemented using the ‘two-stage least squared like’ estimator (TSLS, see Methods).

In the present study we used simulations to compare multiple variance estimators. In addition, an empirical example on the effect of low-density lipoprotein cholesterol (LDL-C) on cardiovascular disease (CVD) is included.

## Methods

### Simulation set-up

Initially we focus on a two-stage meta-analysis where each study has information on a single SNP (Z), a continuous phenotype (X) and a dichotomous endpoint (Y). The goal is to estimate the causal (marginal) odds ratio (OR) of one unit of increase in phenotype on the outcome.

### Data-generating process

J studies were simulated; for the jth study a disease outcome, a phenotype and an IV were generated for $n_{j}$ independent subjects, where j = 1,…, J. To increase readability, the following notation is presented for one study with the same process applied to all studies. The IV variable, Z, counts the number of minor alleles for the ith individual. Following a biallelic model, genotypes were generated from two independent Bernoulli distributions, resulting in the usual Hardy-Weinberg proportions:

$$Prob\left(Z\;=\;0,\;Z\;=\;1,\;Z\;=\;2\right)\;=\;\left(q^{2},\;2pq,\;p^{2}\right).$$

where p represents the probability of the rare allele and q = 1-p the probability of the major allele. Phenotype X was generated dependent on Z and an unobserved confounder C:

$$x_{i}\;=\;\alpha_{0}+\;{\alpha_{1}z_{i}+\alpha }_{2}c_{i}+\;\varepsilon_{i}\;with\;\varepsilon_{i}\;∼\;N\left(0,1\right),\;c_{i}\;∼\;N\left(0,1\right).$$

For the ith individual, the probability of an event was generated based on X and C:

$$logit\left(Prob[y_{i}=1|c_{i},x_{i}]\right)\;=\;log\left(\frac{Prob[y_{i}=1|c_{i},x_{i}]}{1-Prob[y_{i}=1|c_{i},x_{i}]}\right)\;=\;\delta_{0}+\delta_{1}\left(\alpha_{0}+\;{\alpha_{1}z_{i}+\alpha }_{2}c_{i}+\;\varepsilon_{i}\right)+\delta_{2}c_{i}\;=\;\delta_{0}+\delta_{1}x_{i}+\delta_{2}c_{i},\;$$

the event was sampled from a Bernoulli distribution:

$$y_{i}∼Bernoulli\left(Prob\left[y_{i}\;=\;1||c_{i},x_{i}\right]\right).$$

### Data analyses

#### Point estimators

Given that the confounder C is unobserved, it is impossible to estimate the causal effect of the phenotype X on the outcome using regular methods such as logistic regression. Instead, SNP Z can be used to estimate the causal effect of the phenotype on the outcome. The ratio estimator is a relatively straightforward estimator of the logarithm of the causal odds ratio (logOR), which is the estimand here

[1] $$\hat{\theta }\;=\;({\hat{\gamma }}_{1}-{\hat{\delta }}_{3})/{\hat{\alpha }}_{1}.$$

Where ${\hat{\gamma }}_{1}$ represents the effect of the SNP on the outcome measured as the log(OR), ${\hat{\delta }}_{3}$ the log(OR) effect of the SNP on the outcome conditional on the phenotype and unmeasured confounders and ${\hat{\alpha }}_{1}$ the mean difference effect of the SNP on the phenotype (estimated by fitting a linear regression of the type $x_{i}\;=\;{\hat{\alpha }}_{0}+{\hat{\alpha }}_{1}z_{i}+\varepsilon_{i}$ [2]). If every confounding variable (C) *was* measured, ${\hat{\gamma }}_{1}$ and ${\hat{\delta }}_{3}$ could be estimated by fitting the following (logistic regression) models: $logit\left(Prob[y_{i}=1|z_{i}]\right)={\hat{\gamma }}_{0}+{\hat{\gamma }}_{1}z_{i}$ and $logit\left(Prob[y_{i}=1|z_{i},x_{i},c_{i}]\right)={\hat{\delta }}_{0}+{\hat{\delta }}_{1}x_{i}+{\hat{\delta }}_{2}c_{i}+{\hat{\delta }}_{3}z_{i}$. However, because it is never known if all confounders are measured (and correctly specified), this strategy is not feasible. Instead, following the exclusion restriction (assumption ii above), we assume that ${\hat{\delta }}_{3}\;=\;0$, and equation 1 reduces to the ratio of ${\hat{\gamma }}_{1}$ and ${\hat{\alpha }}_{3}$. This ratio estimator is typically used when there is a single instrument or when a multi-gene score is based on a meta-analysis of aggregated results.^12^

Instead of the ratio estimator, the ‘two-stage least squares like’ point estimator (TSLS), also referred to as the two-stage predictor substitution estimators,^13^ is used to estimate the IV effect using a (weighted) gene score.^8^

[3] $$logit(Prob\left[y_{i}\;=\;1||{\hat{x}}_{i}\right])\;=\;{\hat{\beta }}_{0}+\;\hat{\theta }{\hat{x}}_{i}$$

where ${\hat{x}}_{i}$ represents the fitted value of a linear model regressing $x_{i}$ on $z_{i}$ (i.e. the fitted values from a linear regression defined in equation 2).

#### Variance estimators

Following the usual research practice, we will focus on a two-stage meta-analysis where in the second stage study specific results are pooled by the inverse of the variance.^14^ Because results are pooled by the inverse of the variance, we initially focus on different variance estimators, excluding methods that directly estimate a confidence interval.

The delta method^15^^,^^16^ (DM) has the closed form solution:

[4] $${\hat{\sigma }}_{DM}^{2}\;=\;\frac{{\hat{\sigma }}_{\gamma_{1}}^{2}}{{\left({\hat{\alpha }}_{1}\right)}^{2}}+{\hat{\sigma }}_{\alpha_{1}}^{2}\frac{{\left({\hat{\gamma }}_{1}\right)}^{2}}{{\left({\hat{\alpha }}_{1}\right)}^{4}}-2{\hat{\sigma }}_{\gamma_{1},\alpha_{1}}^{2}\frac{{\hat{\gamma }}_{1}}{{\left({\hat{\alpha }}_{1}\right)}^{3}}.$$

Where ${\hat{\sigma }}_{\gamma_{1}}^{2}$ represents the estimated variance in ${\hat{\gamma }}_{1}$, ${\hat{\sigma }}_{\alpha_{1}}^{2}$ the variance in ${\hat{\alpha }}_{1}$ and ${\hat{\sigma }}_{\gamma_{1},\alpha_{1}}^{2}$ the estimated covariance between ${\hat{\gamma }}_{1}\;$and ${\hat{\alpha }}_{1}$. Often the delta method is applied to meta-analysis settings where ${\hat{\sigma }}_{\gamma_{1},\alpha_{1}}^{2}$ is set to zero, resulting in a small overestimation of the variance; this was followed here. Two versions of the delta method were compared: (i) calculating the ratio estimator and the ${\hat{\sigma }}_{DM}^{2}$ in each study followed by meta-analysis of $\hat{\theta }$(DM1); and (ii) calculating $\hat{\theta }\;$using the ratio estimator and ${\hat{\sigma }}_{DM}^{2}$ after separately meta-analysing ${\hat{\gamma }}_{1}$and ${\hat{\alpha }}_{1}$ (DM2).

Alternatively, by sampling with replacement from the observed sample, creating a resampled dataset of size n and repeating this B times, a non-parametric bootstrapped distribution^17^ can be constructed. This distribution can be used to estimate the variance in the IV point estimate [basic bootstrap (BB)]:

[5] $${\hat{\sigma }}_{Boot}^{2}\;=\;\frac{1}{B-1}\sum_{b\;=\;1}^{B}{\left({\bar{\theta }}^{*}-{\hat{\theta }}_{b}^{*}\right)}^{2}\;$$

with ${\hat{\theta }}_{b}^{*}$ the IV estimate estimated in the bth bootstrap sample and ${\bar{\theta }}^{*}$ the mean IV estimate over the B bootstrap samples; here B = 1,000.

All bootstrap variance estimators assume symmetry in bootstrap distribution, due to data sparseness, extreme values of ${\hat{\theta }}_{}^{*}\;$may occur, overestimating the ${\hat{\sigma }}_{Boot}^{2}$. Straightforward solutions that are less sensitive to data sparseness include a bootstrap stratified for the outcome [outcome stratified (OS)] or stratified for the SNP status [SNP stratified (SS)]. A more computer-intensive solution is to perform a double bootstrap (DB)^17^ where for every bth bootstrap sample, R new bootstrap samples of size n are taken using the bth bootstrap sample as the source population. For every bth bootstrap sample the variance is estimated, with the median of these estimates representing the DB IV variance estimate. In our simulations, R = 50 and $B_{DB}\;=\;R*5$. An jack-knife (JK)^17^ variance estimator can also be used:

$${\hat{\sigma }}_{jack}^{2}\;=\;\frac{n\;-1}{n\;}\sum_{i\;=\;1}^{n\;}{\left({\bar{\theta }}_{jack}-{\hat{\theta }}_{-i}\right)}^{2}\;$$

here ${\bar{\theta }}_{jack}\;$represents the mean IV estimate over the n jack-knife estimates and ${\hat{\theta }}_{-i}$ the IV estimate deleting the ith observation.

The previous variance estimators were all applied using the ratio estimator. The robust sandwich (RB) heteroscedasticity-consistent (HC) variance estimator can be used for the TSLS IV, in which the variance estimate ${\hat{\sigma }}_{\hat{x}y}^{2}$ for $\hat{\theta }$ (equation 3) is replaced by the RB estimate. Here we used HC1 and note that JK and RB estimators are related in the sense that the JK approximates the HC3 estimator, which is a refinement of HC1.^18^ Note that the HC estimators are implemented not to adjust for any heteroscedasticity, but merely to penalize the naive variance estimator which assumes that the $\hat{x}$ in equation 3 is measured without error.

### Simulation scenarios

In all simulations J = 10 studies were generated, with $n_{j}$ sampled from a uniform distribution (400, 3600) (see Table 1 for an overview of the simulation parameters). In scenario I, the minor allele frequency (p) was set to 0.50, 0.10, 0.05, 0.01, and 0.005. The probability of the outcome was 0.50. To (initially) prevent weak instrument bias,^19^ the SNP effect on the phenotype was set to $\alpha_{1}\;=\;0.50$, and the unmeasured confounder effect to $\alpha_{2}\;=\;1.00$. By fixing the SNP-phenotype association and decreasing p, the explained variance due to the SNP decreased, as well as the F-statistic. For example, in scenario I the average F-statistic was 126, 46, 25, 6 and 5. To simulate a large amount of confounding, the log(OR) of the unmeasured confounder effect on the outcome was set to $\delta_{2}\;=\;1.50$, the phenotype log(OR) was set to $\delta_{1}\;=\;0.00$ (i.e. no causal effect). In scenario II, p was set to 0.15 and the probability of the outcome was set to 0.10, 0.05, 0.02 and 0.01. Scenarios III and IV differed from II only with respect to p = {0.05, 0.01}.

**Table 1.** Simulation scenarios assessing performance of different variance estimators for an instrumental variable analysis

Parameters	Scenario I	Scenario II	Scenario III	Scenario IV
Number of studies J	10	10	10	10
Sample size sampled from a uniform distribution U(a, b)	(400, 3600)	(400, 3600)	(400, 3600)	(400, 3600)
Minor allele frequency p	{0.50, 0.10, 0.05, 0.01, 0.005}	**0.15**	**0.05**	**0.01**
Effect of SNP on the phenotype $\alpha_{1}$	0.50	0.50	0.50	0.50
Effect of unobserved confounder on the phenotype $\alpha_{2}$	1.00	1.00	1.00	1.00
Intercept $\alpha_{0}$	0.10	0.10	0.10	0.10
Log(OR) of the phenotype effect on the outcome $\delta_{1}$	0.00	0.00	0.00	0.00
Log(OR) of the unobserved confounder effect on the outcome $\delta_{2}$	1.50	1.50	1.50	1.50
Probability of the outcome	0.50	**{0.10, 0.05, 0.02, 0.01}**	{0.10, 0.05, 0.02, 0.01}	{0.10, 0.05, 0.02, 0.01}
Ln(odds) outcome intercept $\delta_{0}$	0.00	**{-2.20, -2.94, -3.89, -4.60}**	{-2.20, -2.94, -3.89, -4.60}	{-2.20, -2.94, -3.89, -4.60}

Changes from the previous scenario (on the left) are presented in bold. Alphas represent mean differences, betas the natural logarithm of the odds ratio.

All simulations were repeated 2000 times and were performed with the statistical package R version 3.1.2 for Unix.^20^ The number of replications was chosen to ensure sufficient precision to detect small deviations from the nominal coverage rate of 0.95 (the 95% lower and upper bounds are 0.940 and 0.960).^21^ Results were pooled using the inverse variance method following a fixed or random effects model where appropriate.

### Performance metrics

Results were evaluated using the following metrics. Mean bias$\left(\bar{\log OR}-log[True\;OR]\right)$, with the first term representing the mean of the $log\hat{OR}$; mean standard error (SE), empirical SE (ESE); estimated by taking the standard deviation of the distribution of $log\hat{OR}$. The root mean squared error $\left[RMSE\;=\;\sqrt{{\left(\bar{logOR}-\log\left[True\;OR\right]\right)}^{2}+{ESE}^{2}}\right]$, coverage rate, defined as the proportion of times the 95% confidence interval (CI) included the true OR, and the number of models that failed to return estimates.

### Additional analyses

Obviously, the absolute performance of the methods depends on the mean sample size per study. To explore the performance in a larger sample size setting, a ‘medium’-sized meta-analysis of 60 000 subjects was simulated by repeating scenario I.

Instead of combining study results in a two-stage meta-analysis, one can also combine datasets in a one-stage meta-analysis. This was explored by repeating scenario I, concatenating the studies together in a single file and adjusting all analyses for study (i.e. bootstrapped by study or adding a study covariable). Given that results do not have to be pooled in a second stage, we only report on a single DM estimator. We instead report on the bootstrap-based percentile confidence interval^22^ which directly estimates the confidence interval (instead of the variance).

In a third sensitivity analysis, scenario I was repeated introducing between-study variance of the gene-phenotype association. This was simulated by replacing $\alpha_{0},\;\alpha_{1}$ and $\varepsilon_{i}$ by $\alpha_{0j}∼N(0.10,\;1^{2})$, $\alpha_{1j}∼N(0.50,\;1^{2})$ and $\varepsilon_{ij}∼N(0,\;\varsigma_{j}^{2})$ with $\varsigma_{j}^{2}∼N(1.50,\;0.3^{2})$.

In a fourth sensitivity analysis, we evaluated the performance of (i) using only the first term of the delta method (the Toby Johnson [TJ] method), and (ii) replacing the asymptotic variance estimates, ${\hat{\sigma }}_{\gamma_{1}}^{2},\;$and ${\hat{\sigma }}_{\alpha_{1}}^{2},$ in the delta method (using the first two terms) by bootstrapped estimates [DM BB]. Both methods were implemented by applying the algorithms before meta-analysis and after meta-analysis (i.e, TJ1, TJ2, DM1 BB, and DM2 BB). Performance was evaluated in scenario I. Additionally, in a fifth sensitivity analysis, we explored performance for continuous outcomes; implemented by repeating scenario I using the parameters of scenario I as mean differences; see Appendix Figure 1 for a flowchart of the methods evaluated (available as Supplementary data at *IJE* online).

## Results

**Figure 1.**
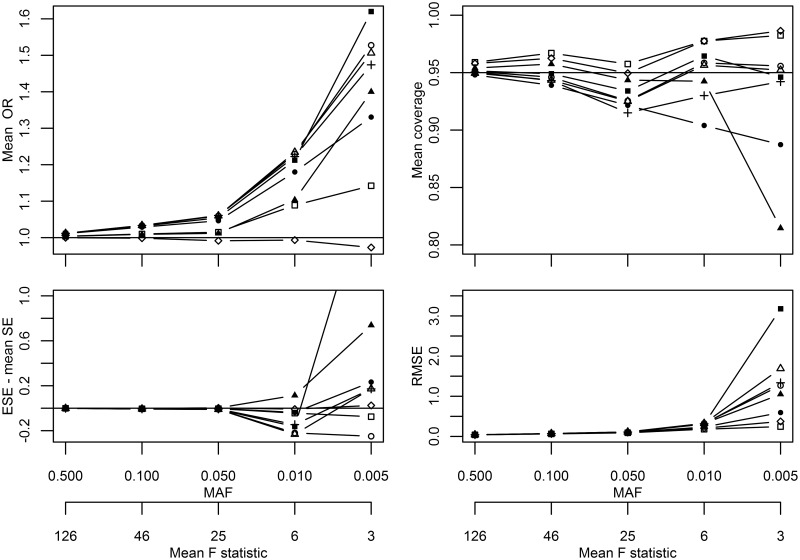
Simulation results from scenarios I comparing different IV variance estimators. *Solid line with a square symbol, delta method followed by meta-analysis [DM1]; solid line with a circle symbol, basic bootstrap [BB]; solid line with triangle symbol, outcome-stratified bootstrap [OS]; solid line with a plus symbol, SNP-stratified bootstrap [SS]; solid line with a filled-out square symbol, double bootstrap [DB]; solid line with a filled-out circle symbol, jack-knife estimator [JK]; solid line with a filled-out triangle symbol, robust variance estimator [RB]; solid line with a rhombus (diamond) symbol, meta-analysis followed by delta method [DM2]. The DB y-value of 2.071 is not depicted for an MAF of 0.005 on the bottom left graph.

Figure 1 depicts the performance of the IV variance estimators under different minor allele frequencies (MAF) or instrument strengths (F-statistic). Unless explicitly stated, all results pertain to the two-stage meta-analysis. At a MAF of 0.50, pooled odds ratio (OR) estimates of all methods were unbiased, but differences between the estimators increased as MAF decreased to 0.005 (or F-statistic went towards zero). Coverage of both the DM estimators increased towards 1.00 as MAF decreased; the RMSE was equal for both DM estimators, and smaller than the RMSE of other methods (Figure 1). JK and RB coverage deteriorated towards 0.80 at lower MAFs. Coverage of the bootstrap methods decreased below 0.95 at a MAF of 0.10/F-statistic 25, recovering to 0.95 at lower MAFs using the BB, SS and DB methods. This unexpected behaviour in coverage was due to the bias in SE (i.e. difference between mean SE and ESE, see Figure 1; Appendix table 1, available as Supplementary data at *IJE* online) trailing behind the bias in OR. Generally the mean SE and ESE agreed well for the DM.

In scenarios II-IV the outcome incidence varied from 0.10 to 0.01 and the MAF was set to 0.15, 0.05 or 0.01, respectively (Appendix Tables 2-4, available as Supplementary data at *IJE* online). At lower outcome probabilities, bias in both DM1 and DM2 was similar, and lower than bias of other methods. For example, in scenario IV at an outcome probability of 0.05, the mean OR was 1.339 and 1.572 for DM1 and DM2, respectively. Coverage of DM1 and DM2 differed substantially at lower outcome probabilities; for example in scenario IV with an outcome probability of 0.01, coverage was 0.793 and 0.550, respectively. Differences between ESE and mean SE were similar however (DM1: -5.729 and DM2: -5.404, respectively), as were the RMSE estimates (DM1: 3.268 and DM2: 3.670, respectively). Coverage of the JK and bootstrap methods was similar and decreased below 0.95 at lower outcome probabilities. RMSE was also similar for all resampling methods, and higher than the DM methods. RB estimates were the most biased, with the lowest coverage and highest RMSE; this coincided with frequent failure of this method to return estimates.

**Figure 2.**
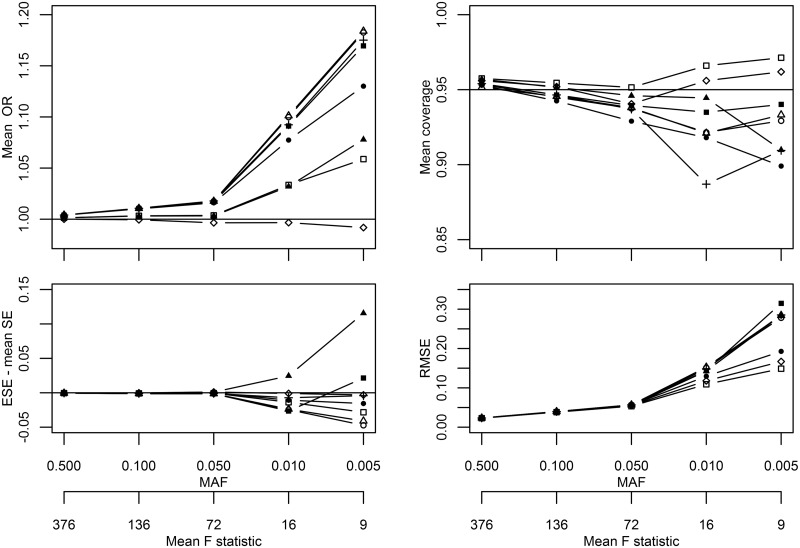
Sensitivity analysis repeating simulation I comparing different IV variance estimators with an average of 60 000 subjects. *Solid line with a square symbol, delta method followed by meta-analysis [DM1]; solid line with a circle symbol, basic bootstrap [BB]; solid line with triangle symbol, outcome-stratified bootstrap [OS]; solid line with a plus symbol, SNP-stratified bootstrap [SS]; solid line with a filled-out square symbol, double bootstrap [DB]; solid line with a filled-out circle symbol, jack-knife estimator [JK]; solid line with a filled-out triangle symbol, robust variance estimator [RB]; solid line with a rhombus (diamond) symbol, meta-analysis followed by delta method [DM2].

**Figure 3.**
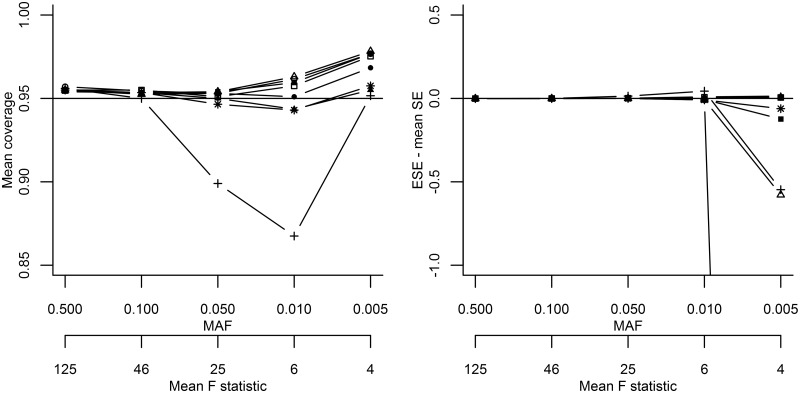
Sensitivity analysis repeating simulation I comparing different IV variance estimators using a one-stage meta-analysis design with an average of 20 000 subjects. *Solid line with a square symbol, delta method followed by meta-analysis [DM1]; solid line with a circle symbol, basic bootstrap [BB]; solid line with triangle symbol, outcome-stratified bootstrap [OS]; solid line with a plus symbol, SNP-stratified bootstrap [SS]; solid line with a filled-out square symbol, double bootstrap [DB]; solid line with a filled-out circle symbol, jack-knife estimator [JK]; solid line with a filled-out triangle symbol, robust variance estimator [RB]; solid line with a star symbol, bootstrapped percentile method. The BB y-value of -13.463 is not depicted for an MAF of 0.005 on the right graph.

Repeating scenario I with a larger sample size (60 000 subjects) showed a comparable relative pattern as before (Figure 2; Appendix Table 5, available as Supplementary data at *IJE* online). Repeating scenario I using a one-stage meta-analysis (20 000 subjects) improved performance. There was no difference between the methods in mean OR, bias or RMSE (Appendix Table 6, available as Supplementary data at *IJE* online); even in extreme settings, bias was low at -0.016 (MAF of 0.005 or F-statistic of 4). Coverage (Figure 3) was generally close to 0.95 or slightly larger, and agreement between mean SE and ESE was generally good, only deviating at a MAF of 0.005 or an F-statistic of 4. A non-parametric bootstrap percentile confidence interval was evaluated, performing similarly to other methods (coverage ≈ 0.95). Repeating scenario I with between-study variance showed similar performance as in the original fixed effect scenario (Appendix Table 7, available as Supplementary data at *IJE* online), except for more conservative coverage rates and DM2 being the most biased estimator at MAF ≤ 0.01, e.g. -0.257 mean bias at MAF 0.005, which coincided with a coverage rate of almost 1, and a RMSE of 10.289. DM1 performed better than other methods with a coverage of 0.981 and an RMSE of 0.127, at a MAF of 0.005.

The Toby Johnson [TJ] variance estimator performed comparably to the DM1 or DM2 in scenario I with only slightly lower coverage (Appendix Table 8, available as Supplementary data at *IJE* online). Implementing the delta method by replacing the asymptotic variance estimators with bootstrapped estimators [DM BB] performed similarly to the BB method (Appendix Table 8). Repeating scenario I with a continuous outcome revealed a comparable relative performance of the variance estimators (Appendix Table 9, available as Supplementary data at *IJE* online).

### The LDL-C effect on CVD

**Figure 4.**
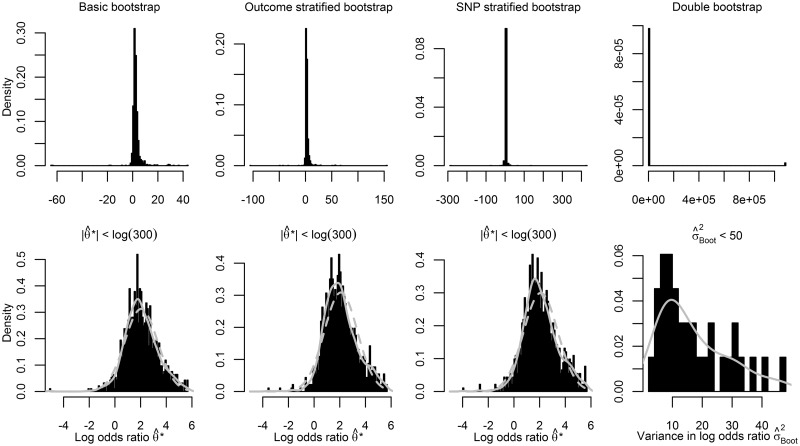
Bootstrap distributions for IV rs2965101 for the relation of LDL-C and CVD. *Solid grey lines indicate the non-parametric density (only presented in the second row), with dashed grey lines indicating the expected density given a normal distribution (not presented for the double bootstrap).

Table 2 shows empirical results of two different IVs in a six-study meta-analysis to estimate the effect of LDL-C on CVD (see Appendix at *IJE* online for a description of the data sources, and baseline data). Using SNP rs11591147 as an IV (mean F-statistic = 13.42) in a two-stage meta-analysis showed that the bootstrap methods had the largest standard errors and their point estimates not only disagreed with results from the remaining variance estimators but also between themselves. As expected, using a one-stage meta-analysis increased precision and decreased differences between methods, resulting in an IV estimate of 0.93 (95% CI 0.50;1.72). Results from the weak instrument rs2965101 (mean F-statistic = 1.34) revealed large differences between the bootstrap estimators and the remaining estimators; the minimal bootstrap SE estimate was 13.19, compared with an SE of 1.49 using DM2. Precision increased using a one-stage meta-analysis, however the bootstrapped SE were still comparatively large. Given that one-stage meta-analyses are analysed by a single analyst, it becomes practical to explore the bootstrap distributions (Figure 4). After omitting a number of outliers, the bootstrap became relatively symmetrical and the SE estimates were: 1.27 (BB), 1.29 (OS), 1.33 (SS) and 3.51 (DB). The large SE of the DB and its truncated distribution show that 50 times 250 repetitions were insufficient in this setting.

**Table 2.** Instrumental variable analysis of the LDL-C effect on CVD using instrument rs11591147 or rs2965101

	**Fixed effect two-stage meta-analysis**		**Random effects two-stage meta-analysis**		**Fixed effect one-stage meta-analysis**		**Heterogeneity statistics**	
	Odds ratio (95% CI)	SE	Odds ratio (95% CI)	SE	Odds ratio (95% CI)	SE	*˙Χ^2^*(*P*-value)	*τ^2^*
**Crude LDL-C association**	1.06(1.01;1.11)	0.03	1.10(0.96;1.25)	0.07	1.06(1.01;1.11)	0.03	33.25(0.00)	0.02
**rs11591147 IV LDL-C estimates**								
DM before MA [DM1]	0.94(0.50;1.75)	0.32	094(0.50;1.75)	0.32	0.93(0.50;1.72)	0.31	4.88(0.43)	0.00
Basic bootstrap [BB]	1.24(0.48;3.18)	0.48	1.24(0.48;3.18)	0.48	0.93(0.49;1.76)	0.33	0.98(0.98)	0.00
Outcome-stratified bootstrap [OS]	1.44(0.49;4.18)	0.55	1.44(0.49;4.18)	0.55	0.93(0.49;1.78)	0.33	0.08(1.00)	0.00
SNP-stratified bootstrap [SS]	0.89(0.30;2.64)	0.55	0.89(0.30;2.64)	0.55	0.93(0.50;1.72)	0.31	0.38(1.00)	0.00
Double bootstrap [DB]	1.05(0.38;2.85)	0.51	1.05(0.38;2.85)	0.51	0.93(0.50;1.72)	0.31	1.58(0.93)	0.00
Jack-knife [JK]	0.90(0.45;1.81)	0.35	0.90(0.45;1.81)	0.35	0.93(0.51;1.69)	0.31	4.05(0.58)	0.00
Robust HC1 [RB]	0.82(0.45;1.51)	0.31	0.81(0.41;1.60)	0.35	0.93(0.50;1.74)	0.32	5.85(0.33)	0.11
DM after MA [DM2]	0.87(0.46;1.65)	0.33	0.85(0.40;1.80)	0.38	NA	NA	7.47(0.19)/6.21 (0.29)	0.01/0.03
Percentile method	NA	NA	NA	0.00	0.93(0.49;1.78)	NA	NA	NA
**rs2965101 IV LDL-C estimates**								
DM before MA [DM1]	1.55(0.35;17.90)	1.25	1.55(0.13;17.90)	1.25	8.16(0.50;132.64)	1.42	3.11(0.66)	0.00
Basic bootstrap [BB]	0.61(0.00;2*10^21^)	25.35	0.61(0.00;2*10^21^)	25.35	8.16(0.00;9*10^4^)	4.77	0.01(1.00)	0.00
Outcome-stratified bootstrap [OS]	4.61(0.00;5*10^30^)	35.32	4.61(0.00;5*10^30^)	35.32	8.16(0.00;6*10^7^)	8.07	0.01(1.00)	0.00
SNP stratified bootstrap [SS]	6.67(0.00;10^29^)	33.29	6.67(0.00;10^29^)	33.29	8.16(0.00;4*10^15^)	17.21	0.00(1.00)	0.00
Double bootstrap [DB]	1.55(0.00;3*10^11^)	13.19	1.55(0.00;3*10^11^)	13.19	8.16(0.00;10^5^)	4.93	0.03(1.00)	0.00
Jack-knife [JK]	1.56(0.13;18.04)	1.25	1.56(0.13;18.04)	1.25	8.16(0.70;95.04)	1.25	3.13(0.65)	0.00
Robust HC1 [RB]	3.03(0.47;19.47)	0.95	2.72(0.20;37.48)	1.34	8.16(0.91;72.85)	1.12	8.11(0.13)	3.86
DM after MA [DM2]	8.52(0.46;157.69)	1.49	9.01(0.36;223.27)	1.64	NA	NA	2.64(0.76)/6.14 (0.29)	0.00/0.00
Percentile method	NA	NA	NA	NA	8.16(0.88;10^5^)	NA	NA	NA

MA, meta-analysis; NA, not available.

The mean F-statistics of the two-stage designed IPDMAs were 13.42, and 1.34 for rs11591147 and rs2965101, respectively. The F-statistics of the one-stage designed IPDMA were 500.07, and 485.53, rs11591147 and rs2965101, respectively. The explained variance due to the instruments (measured as the squared Spearman correlation coefficient) were 0. 70*10^-2^ and 0. 64*10^-4^. The heterogeneity statistics were determined for the fixed effect two-stage meta-analysis, tau-squared was calculated using the methods of moments estimator, chi-squared test statistic and *P*-value were based on the Q-test. The percentile method is only available for the one-stage design. For DM2, the heterogeneity statistics represent the heterogeneity in ${\hat{\alpha }}_{1}$ and $\hat{\gamma }$_1, see equation 1.

## Discussion

This study showed that, depending on the strength of the IV and/or the outcome incidence, there is considerable difference in the performance of instrumental variable (IV) variance estimators in two-stage meta-analysis. The delta method (DM) showed the least amount of bias and the best coverage, with the delta method implemented before meta-analysis performing better in the presence of between-study variance. Bootstrap and robust variance estimators (RB) produced extreme estimates in two-stage meta-analysis. Differences between methods were minimal using a one-stage meta-analysis, all providing unbiased estimates and appropriate coverage. An empirical example on the LDL-C effect on CVD incidence confirmed that these issues also occur in applied settings. Relative performance of the variance estimators was similar when using a continuous outcome instead of a binary endpoint.

At lower MAF/F-statistic values or lower outcome probabilities, the RB estimators often failed to converge, making it difficult to evaluate whether the underperformance of RB was due to the estimator itself or to informative failures. Looking at the JK (which failed in less than 1% of the simulations, and which is an approximation of the HC3; which is a refinement of the HC1 used in the RB), it seems that to some extent this underperformance of the RB may be explained by computational problems in the R sandwich package.^23^ Following the usual practice in applied Mendelian randomization analyses, the ratio and the TSLS point estimators were used. Additionally to the usual three IV assumptions, these point estimators also assume the phenotype to be normally distributed conditional on the SNP and confounders and homogeneity of the phenotype (X) effect on the outcome.^24^ In our simulations these assumptions held; however in applied settings this is not necessarily the case, given that confounders are often unmeasured these assumptions are also impossible to evaluate. Instead of making these assumptions, different estimators or estimands may be considered in empirical settings. For example, structural mean models, or generalized method of moments point estimators or the risk difference estimand^8^^,^^24^ make fewer assumptions.

Our results underline the difficulty of using the observed F-statistic^7^ as a measure of expected bias due to a weak instrument. We observed an increased performance in a one-stage meta-analysis with on average 20 000 subjects and a ‘weak’ instrument (MAF 0.05, mean F-statistic 5.97), compared with a two-stage meta-analysis with on average 60 000 subjects and a ‘strong’ instrument (MAF 0.05, mean F-statistic 15.98). When conducting a one-stage meta-analysis, results do not have to be pooled by the inverse of an estimated study-specific variance. Therefore in this scenario, point estimates, precision (ESE) and RMSEs were not influenced by the choice of variance estimators. The choice of variance estimator did influence coverage, which was nevertheless markedly improved over a two-stage design.

The underperformance of the bootstrap estimators in the two-stage meta-analysis may come as a surprise to some; however, the improved performance (over for example a Wald-based confidence interval) shown in the literature mostly holds for bootstrap confidence intervals such as the bias corrected and accelerated bootstrapped confidence interval.^17^^,^^22^^,^^25^ Because of the need for a variance estimate in the second stage of a two-stage meta-analysis, the bootstrap can only be used to estimate the standard error of the IV estimate, which implicitly assumes symmetry of the bootstrap distribution.^17^^,^^22^^,^^25^ We did however evaluate the percentile method to directly estimate the confidence interval when we replicated scenario I using a one-stage meta-analysis. Results indeed showed proper coverage; however, this was similar to the increased performance of all other estimators. We evaluated a delta method estimator replacing the asymptotic variance estimates by bootstrapped variance estimates; this approach performed worse than the regular delta method (DM1 or DM2). These results show that even though the asymptotic approximations of$\;{\hat{\sigma \;}}_{\gamma_{1}}^{2}\;$and ${\hat{\sigma }}_{\alpha_{1}}^{2}$ do not strictly hold, these estimates are better approximations (in such situations) than bootstrapped alternatives.

The simulations presented here are naturally limited and the following points merit discussion. First, different simulation parameters will result in different absolute performance. Instead, we focused on relative (i.e. between methods) performance which we expect to be more robust. Second, by fixing the effect of the instrument (the SNP) on the phenotype, the instrument strength decreases with MAF; hence our results include analyses with F-statistics below 10. These are analyses, some might argue, an applied researcher would not perform due to violation of IV assumption 1. We showed, however, that despite the ‘weak’ instrument, valid estimates can be derived. Third, although it seems logical to increase the number of bootstraps as the data become sparser (or the IV becomes weaker), we kept the number fixed to preserve comparability between scenarios. Fourth, for simplicity we focused on scenarios with a single SNP instrument whereas, to prevent weak-instrument bias, most Mendelian randomization studies use multiple SNPs. Nevertheless, relevant information for these multiple SNP approaches can be found in our analyses by focusing on strong-instrument settings. Fifth, we only explored performance under the null [i.e. OR = 1] because (i) coverage was often too low, making comparisons on power pointless, and (ii) we wished to prevent influence of non-collapsibility.^26^ Sixth, the small ORs observed in low-frequency scenarios were most likely due to the outcome being constant for a certain allele number (i.e. perfect separation). In these settings, penalized models, using for example a Firth^27^^,^^28^ or Lasso^29^ penalization, are expected to perform better.^30^ Finally, random effects or fixed effect analysis models were used depending on whether the simulation scenario included between-study variance or not.^31^ In empirical analyses, the choice between random effects and fixed effect models typically depends on a heterogeneity measure.^32^ However, bias in point and variance estimates will influence the observed heterogeneity, resulting in different modelling choices depending on the performance of the estimator. This would make between-methods comparisons difficult. Therefore, the choice of model was based on the true, rather than the observed, between-study variance.

In conclusion, the choice of variance estimator in instrumental variable analyses using a two-stage meta-analysis is important. Simulations showed that the delta method applied at stage one of the two-stage meta-analysis performed best. If resampling variance estimators are used, we suggest always checking study-specific plots of these distributions for outliers. This is especially important if the outcome and/or SNPs are rare or if the instrument is weak. Out of all the resampling methods, the jack-knife estimator performed best. However, in such a scenario an even better alternative, when possible, is to perform a one-stage meta-analysis making the choice of variance estimator less influential. If a one-stage design is used, resampling techniques can be used to directly estimate confidence intervals for which methods exist that do not assume a symmetrical distribution (e.g. the percentile method).

## Funding

A.F.S. and A.D.H. are funded by UCLH NIHR Biomedical Research Centre (BRC10200). F.D. is funded by the MRC (K006215). A.F.S. is additionally funded by a UCL springboard population science fellowship. The UCLEB Consortium is supported by a British Heart Foundation Programme Grant (RG/10/12/28456). We acknowledge the British Regional Heart Study team for data collection. The British Regional Heart study is supported by British Heart Foundation grants (RG/08/013/25942) and BHF (RG/13/16/30528). The British Heart Foundation had no role in the design or conduct of the study; collection, management, analysis or interpretation of the data; preparation, review or approval of the manuscript; or the decision to submit the manuscript for publication. The WHII study is supported by grants from the Medical Research Council (G0902037; ID85374), British Heart Foundation (RG/07/008/23674), Stroke Association, National Heart Lung and Blood Institute (5RO1 HL036310), National Institute on Aging (5RO1AG13196), Agency for Health Care Policy Research (HS06516) and the John D. and Catherine T. MacArthur Foundation Research Networks on Successful Midlife Development and Socio-economic Status and Health. Samples from the ELSA DNA Repository (EDNAR) received support under a grant (AG1764406S1) awarded by the National Institute on Ageing (NIA). ELSA was developed by a team of researchers based at the National Centre for Social Research, University College London and the Institute of Fiscal Studies. The data were collected by the National Centre for Social Research. CaPS was funded by the Medical Research Council and undertaken by the former MRC Epidemiology Unit (South Wales). The DNA bank was established with funding from an MRC project grant. The data archive is maintained by the University of Bristol. EAS is funded by the British Heart Foundation (Programme Grant RG/98002), with Metabochip genotyping funded by a project grant from the Chief Scientist Office of Scotland (Project Grant CZB/4/672). MRC NSHD is funded by the UK Medical Research Council. The WHII study is supported by grants from the Medical Research Council (G0902037; ID85374), British Heart Foundation (RG/07/008/23674), Stroke Association, National Heart Lung and Blood Institute (5RO1 HL036310), National Institute on Aging (5RO1AG13196), Agency for Health Care Policy Research (HS06516) and the John D. and Catherine T. MacArthur Foundation Research Networks on Successful Midlife Development and Socio-economic Status and Health.

## Supplementary Material

Supplementary Data
