# Supplementary material for: Comparison of variance estimators for meta-analysis of instrumental variable estimates
Source: Int J Epidemiol. 2016 Sep 2;45(6):1975–86. doi: 10.1093/ije/dyw123 (PMC5654757; doi:10.1093/ije/dyw123)
Supplement: Supplementary Data [file dyw123_supplementary_data.zip › ije-2015-09-1226-File011.docx]

**Figure captions**

**Figure 1 Simulation results from scenarios I comparing different IV estimators.***

[Figure 1 here]

* orange dashed line with a square symbol, delta method followed by meta-analysis [DM1]; blue dashed line with a circle symbol, basic bootstrap [BB]; red dashed line with triangle symbol, outcome stratified bootstrap [OS]; green dashed line with a plus symbol, SNP stratified bootstrap [SS]; black dashed dotted line with a filled out square symbol, double bootstrap [DB]; orange dashed dotted line with a filled out circle symbol, jackknife estimator [JK]; blue dashed dotted line with a filled out triangle symbol, robust variance estimator [RB]; red dashed dotted line with a rhombus (diamond) symbol, meta-analysis followed by delta method [DM2]. The DB y-value of 2.071 is not depicted for a MAF of 0.005 on the bottom left graph.

**Figure 2 Sensitivity analysis repeating simulation I comparing different IV estimators with an average of 60,000 subjects.***

[Figure 2 here]

* orange dashed line with a square symbol, delta method followed by meta-analysis [DM1]; blue dashed line with a circle symbol, basic bootstrap [BB]; red dashed line with triangle symbol, outcome stratified bootstrap [OS]; green dashed line with a plus symbol, SNP stratified bootstrap [SS]; black dashed dotted line with a filled out square symbol, double bootstrap [DB]; orange dashed dotted line with a filled out circle symbol, jackknife estimator [JK]; blue dashed dotted line with a filled out triangle symbol, robust variance estimator [RB]; red dashed dotted line with a rhombus (diamond) symbol, meta-analysis followed by delta method [DM2].

**Figure 3 Sensitivity analysis repeating simulation I comparing different IV estimators using a one stage meta-analysis design with an average of 20,000 subjects.***

[Figure 3 here]

* orange dashed line with a square symbol, delta method followed by meta-analysis [DM1]; blue dashed line with a circle symbol, basic bootstrap [BB]; red dashed line with triangle symbol, outcome stratified bootstrap [OS]; green dashed line with a plus symbol, SNP stratified bootstrap [SS]; black dashed dotted line with a filled out square symbol, double bootstrap [DB]; orange dashed dotted line with a filled out circle symbol, jackknife estimator [JK]; blue dashed dotted line with a filled out triangle symbol, robust variance estimator [RB]; red dashed dotted line with a star symbol, bootstrapped percentile method. The BB y-value of -13.463 is not depicted for a MAF of 0.005 on the right graph.

**Figure 4 Bootstrap distributions for IV rs2965101 for the relation of LDL-C and CVD.***

[Figure 4 here]

* Solid grey lines indicate the non-parametric density (only presented in the second row), with dashed grey lines indicating the expected density given a normal distribution (not presented for the double bootstrap).
