# Supplementary material for: Comparison of variance estimators for meta-analysis of instrumental variable estimates
Source: Int J Epidemiol. 2016 Sep 2;45(6):1975–86. doi: 10.1093/ije/dyw123 (PMC5654757; doi:10.1093/ije/dyw123)
Supplement: Supplementary Data [file dyw123_supplementary_data.zip › ije-2015-09-1226-File003.docx]

**Data sources for the empirical example of the LDL-C effect on CVD.**

To empirically compare performance of the different estimators, see main text, we used SNP rs11591147 in the PCSK9 gene and SNP rs2965101 in the BCL3 gene as instruments to estimate the causal effect of LDL-C on CVD. Data were used from 6 studies in the UCLEB consortium(1) (overall n = 11581 with minimal n = 764, and maximum n = 3041; overall CVD events = 2050), British Regional Heat Study (BRHS)(2), Caerphilly Prospective Study (CaPS)(3), Edinburgh Artery Study (EAS)(4), English Longitudinal Study of Ageing (ELSA)(5), MRC National Survey of Health and Development (MRC46)(6), and Whitehall-II (WHII)(7). Between study heterogeneity was measured using the Q-test (8) and the method of moments estimator of the tau-squared (9). These two instruments were chosen because of a lack of pleiotropy (Appendix 1 figure 1), small correlation (r < 0.01), their different frequency (rs11591147 average $p$ = 0.02, min 0.02; max 0.02; rs2965101 average $p$ = 0.32, min 0.31; max 0.33), and different magnitudes of association with LDL-C (Spearman correlations of -0.082 and -0.008 for rs11591147 and rs2965101 with LDL-C).

Appendix table 1 Simulation results for scenario I assessing performance of different instrumental variable variance estimators under different levels of MAF with an outcome probability of 0.50 *.

|  | MAF = 0.500 | MAF = 0.100 | MAF = 0.050 | MAF = 0.010 | MAF = 0.005 |
| --- | --- | --- | --- | --- | --- |
| **Mean odds ratio (truth=1.000)**  Crude  DM before MA [DM1]  Basic bootstrap [BB]  Outcome stratified bootstrap [OS]  SNP stratified bootstrap [SS]  Double bootstrap [DB]  Jackknife [JK]  Robust HC1 [RB]  DM after MA [DM2] | 1.778  1.004  1.012  1.012  1.012  1.011  1.011  1.004  1.000 | 1.826  1.010  1.033  1.033  1.033  1.031  1.029  1.009  0.998 | 1.840  1.015  1.060  1.060  1.061  1.054  1.046  1.012  0.991 | 1.851  1.089  1.229  1.234  1.222  1.212  1.180  1.104  0.993 | 1.853  1.142  1.528  1.506  1.474  1.620  1.331  1.400  0.973 |
| **Mean bias**  Crude  DM before MA [DM1]  Basic bootstrap [BB]  Outcome stratified bootstrap [OS]  SNP stratified bootstrap [SS]  Double bootstrap [DB]  Jackknife [JK]  Robust HC1 [RB]  DM after MA [DM2] | 0.576  0.004  0.012  0.012  0.012  0.011  0.011  0.004  0.000 | 0.602  0.010  0.033  0.033  0.033  0.031  0.028  0.009  -0.002 | 0.610  0.015  0.058  0.058  0.059  0.053  0.045  0.012  -0.009 | 0.616  0.085  0.206  0.211  0.201  0.193  0.166  0.099  -0.007 | 0.617  0.133  0.424  0.410  0.388  0.482  0.286  0.336  -0.027 |
| **Coverage**  Crude  DM before MA [DM1]  Basic bootstrap [BB]  Outcome stratified bootstrap [OS]  SNP stratified bootstrap [SS]  Double bootstrap [DB]  Jackknife [JK]  Robust HC1 [RB]  DM after MA [DM2] | 0.000  0.959  0.951  0.950  0.951  0.952  0.948  0.954  0.958 | 0.000  0.967  0.946  0.944  0.943  0.949  0.939  0.958  0.963 | 0.000  0.958  0.926  0.925  0.915  0.934  0.922  0.944  0.950 | 0.000  0.978  0.959  0.957  0.930  0.965  0.904  0.943  0.978 | 0.000  0.982  0.956  0.952  0.942  0.946  0.887  0.815  0.986 |
| **Mean SE**  Crude  DM before MA [DM1]  Basic bootstrap [BB]  Outcome stratified bootstrap [OS]  SNP stratified bootstrap [SS]  Double bootstrap [DB]  Jackknife [JK]  Robust HC1 [RB]  DM after MA [DM2] | 0.012  0.040  0.041  0.041  0.041  0.041  0.040  0.040  0.040 | 0.012  0.068  0.071  0.071  0.070  0.071  0.067  0.067  0.068 | 0.013  0.093  0.104  0.104  0.101  0.104  0.093  0.092  0.094 | 0.013  0.202  0.472  0.479  0.389  0.400  0.201  0.191  0.212 | 0.013  0.281  1.445  1.466  1.106  1.072  0.288  0.256  0.347 |
| **ESE**  Crude  DM before MA [DM1]  Basic bootstrap [BB]  Outcome stratified bootstrap [OS]  SNP stratified bootstrap [SS]  Double bootstrap [DB]  Jackknife [JK]  Robust HC1 [RB]  DM after MA [DM2] | 0.012  0.040  0.040  0.040  0.040  0.040  0.040  0.040  0.040 | 0.012  0.063  0.064  0.064  0.064  0.064  0.063  0.065  0.066 | 0.013  0.089  0.094  0.094  0.094  0.093  0.091  0.094  0.096 | 0.013  0.159  0.252  0.248  0.244  0.237  0.164  0.306  0.204 | 0.013  0.205  1.196  1.639  1.278  3.143  0.522  0.995  0.372 |
| **RMSE**  Crude  DM before MA [DM1]  Basic bootstrap [BB]  Outcome stratified bootstrap [OS]  SNP stratified bootstrap [SS]  Double bootstrap [DB]  Jackknife [JK]  Robust HC1 [RB]  DM after MA [DM2] | 0.576  0.040  0.041  0.041  0.041  0.041  0.041  0.040  0.040 | 0.602  0.063  0.072  0.072  0.072  0.071  0.069  0.065  0.066 | 0.610  0.091  0.110  0.111  0.111  0.107  0.102  0.095  0.097 | 0.616  0.180  0.325  0.325  0.316  0.305  0.233  0.322  0.205 | 0.617  0.245  1.269  1.689  1.336  3.179  0.595  1.050  0.373 |
| **Number of failed models**  Crude  DM before MA [DM1]  Basic bootstrap [BB]  Outcome stratified bootstrap [OS]  SNP stratified bootstrap [SS]  Double bootstrap [DB]  Jackknife [JK]  Robust HC1 [RB]  DM after MA [DM2] | 0  0  0  0  0  0  0  0  0 | 0  0  0  0  0  0  0  0  0 | 0  0  0  0  0  0  0  0  0 | 0  0  0  0  0  0  0  0  0 | 0  12  12  12  12  12  12  15  12 |
| **ESE – mean SE**  Crude  DM before MA [DM1]  Basic bootstrap [BB]  Outcome stratified bootstrap [OS]  SNP stratified bootstrap [SS]  Double bootstrap [DB]  Jackknife [JK]  Robust HC1 [RB]  DM after MA [DM2] | 0.000  -0.001  -0.001  -0.001  -0.001  -0.001  -0.001  0.000  0.000 | 0.000  -0.005  -0.007  -0.007  -0.007  -0.008  -0.004  -0.002  -0.002 | 0.000  -0.004  -0.010  -0.010  -0.007  -0.011  -0.002  0.003  0.003 | 0.000  -0.044  -0.220  -0.231  -0.146  -0.163  -0.038  0.115  -0.008 | 0.000  -0.075  -0.250  0.173  0.172  2.071  0.233  0.739  0.025 |

* MAF = minor allele frequency; DM = delta method; MA = meta-analysis; SNP = single nucleotide polymorphism ; SE = standard error; ESE = empirical standard error; RMSE = square root of the mean squared error. The crude model regresses the log(odds) of the dichotomous outcome on the continuous phenotype. The mean F-statistics for the IV-phenotype association are: 126.42, 45.97, 24.67, 5.98, 3.47

**Appendix table 2 Simulation results for scenario II assessing performance of different instrumental variable variance estimators under different probabilities for the outcome with the MAF fixed at 0.15. ***

|  | Prob(y = 1) = 0.1 | Prob(y = 1) = 0.05 | Prob(y = 1) = 0.02 | Prob(y = 1) = 0.01 |
| --- | --- | --- | --- | --- |
| **Mean odds ratio (truth=1.000)**  Crude  DM before MA [DM1]  Basic bootstrap [BB]  Outcome stratified bootstrap [OS]  SNP stratified bootstrap [SS]  Double bootstrap [DB]  Jackknife [JK]  Robust HC1 [RB]  DM after MA [DM2] | 1.269  1.010  1.018  1.018  1.018  1.017  1.017  1.010  1.008 | 1.272  1.022  1.034  1.034  1.035  1.033  1.030  1.023  1.019 | 1.273  1.047  1.099  1.099  1.099  1.091  1.066  0.937  1.048 | 1.272  1.105  1.290  1.298  1.293  1.272  1.147  0.494  1.113 |
| **Mean bias**  Crude  DM before MA [DM1]  Basic bootstrap [BB]  Outcome stratified bootstrap [OS]  SNP stratified bootstrap [SS]  Double bootstrap [DB]  Jackknife [JK]  Robust HC1 [RB]  DM after MA [DM2] | 0.238  0.010  0.018  0.018  0.018  0.017  0.016  0.010  0.008 | 0.241  0.021  0.034  0.033  0.034  0.032  0.029  0.023  0.019 | 0.241  0.046  0.095  0.094  0.095  0.087  0.064  -0.065  0.047 | 0.240  0.100  0.254  0.261  0.257  0.241  0.137  -0.706  0.107 |
| **Coverage**  Crude  DM before MA [DM1]  Basic bootstrap [BB]  Outcome stratified bootstrap [OS]  SNP stratified bootstrap [SS]  Double bootstrap [DB]  Jackknife [JK]  Robust HC1 [RB]  DM after MA [DM2] | 0.000  0.965  0.964  0.964  0.963  0.965  0.961  0.953  0.959 | 0.000  0.958  0.958  0.956  0.958  0.962  0.953  0.948  0.949 | 0.000  0.958  0.949  0.952  0.952  0.960  0.954  0.905  0.949 | 0.002  0.945  0.913  0.894  0.910  0.925  0.936  0.720  0.937 |
| **Mean SE**  Crude  DM before MA [DM1]  Basic bootstrap [BB]  Outcome stratified bootstrap [OS]  SNP stratified bootstrap [SS]  Double bootstrap [DB]  Jackknife [JK]  Robust HC1 [RB]  DM after MA [DM2] | 0.016  0.091  0.095  0.094  0.094  0.095  0.092  0.090  0.091 | 0.022  0.124  0.130  0.129  0.130  0.132  0.125  0.122  0.124 | 0.034  0.193  0.217  0.211  0.216  0.219  0.199  0.189  0.194 | 0.047  0.272  0.381  0.345  0.380  0.374  0.289  0.258  0.273 |
| **ESE**  Crude  DM before MA [DM1]  Basic bootstrap [BB]  Outcome stratified bootstrap [OS]  SNP stratified bootstrap [SS]  Double bootstrap [DB]  Jackknife [JK]  Robust HC1 [RB]  DM after MA [DM2] | 0.016  0.087  0.087  0.087  0.087  0.087  0.087  0.089  0.090 | 0.021  0.118  0.120  0.120  0.120  0.120  0.119  0.122  0.123 | 0.034  0.182  0.192  0.187  0.191  0.191  0.184  0.848  0.188 | 0.048  0.259  0.344  0.305  0.333  0.324  0.271  2.308  0.265 |
| **RMSE**  Crude  DM before MA [DM1]  Basic bootstrap [BB]  Outcome stratified bootstrap [OS]  SNP stratified bootstrap [SS]  Double bootstrap [DB]  Jackknife [JK]  Robust HC1 [RB]  DM after MA [DM2] | 0.239  0.087  0.089  0.089  0.089  0.089  0.088  0.089  0.090 | 0.242  0.120  0.125  0.124  0.124  0.124  0.123  0.124  0.124 | 0.244  0.188  0.214  0.210  0.213  0.210  0.195  0.850  0.194 | 0.245  0.277  0.428  0.402  0.420  0.404  0.303  2.413  0.286 |
| **Number of failed models**  Crude  DM before MA [DM1]  Basic bootstrap [BB]  Outcome stratified bootstrap [OS]  SNP stratified bootstrap [SS]  Double bootstrap [DB]  Jackknife [JK]  Robust HC1 [RB]  DM after MA [DM2] | 0  0  0  0  0  0  0  0  0 | 0  0  0  0  0  0  0  0  0 | 0  0  0  0  0  0  0  6  0 | 0  0  0  0  0  0  0  39  0 |
| **ESE – mean SE**  Crude  DM before MA [DM1]  Basic bootstrap [BB]  Outcome stratified bootstrap [OS]  SNP stratified bootstrap [SS]  Double bootstrap [DB]  Jackknife [JK]  Robust HC1 [RB]  DM after MA [DM2] | 0.000  -0.005  -0.007  -0.007  -0.007  -0.008  -0.005  -0.001  -0.002 | -0.001  -0.006  -0.010  -0.010  -0.010  -0.011  -0.006  -0.001  -0.001 | 0.000  -0.011  -0.024  -0.024  -0.025  -0.028  -0.015  0.659  -0.006 | 0.001  -0.014  -0.037  -0.039  -0.047  -0.050  -0.019  2.050  -0.008 |

* MAF = minor allele frequency; DM = delta method; MA = meta-analysis; SNP = single nucleotide polymorphism ; SE = standard error; ESE = empirical standard error; RMSE = square root of the mean squared error. The crude model regresses the log(odds) of the dichotomous outcome on the continuous phenotype. The mean F-statistic for the IV-phenotype association is 64.62.

**Appendix table 3 Simulation results for scenario III assessing performance of different instrumental variable variance estimators under different probabilities for the outcome with the MAF fixed at 0.05. ***

|  | Prob(y = 1) = 0.10 | Prob(y = 1) = 0.05 | Prob(y = 1) = 0.02 | Prob(y = 1) = 0.01 |
| --- | --- | --- | --- | --- |
| **Mean odds ratio (truth=1.000)**  Crude  DM before MA [DM1]  Basic bootstrap [BB]  Outcome stratified bootstrap [OS]  SNP stratified bootstrap [SS]  Double bootstrap [DB]  Jackknife [JK]  Robust HC1 [RB]  DM after MA [DM2] | 1.276  1.037  1.063  1.062  1.065  1.062  1.055  1.025  1.034 | 1.278  1.062  1.126  1.125  1.131  1.120  1.093  0.810  1.068 | 1.279  1.195  1.540  1.551  1.547  1.490  1.277  0.038  1.227 | 1.277  1.412  0.206  0.304  0.178  0.158  1.238  <0.001  1.490 |
| **Mean bias**  Crude  DM before MA [DM1]  Basic bootstrap [BB]  Outcome stratified bootstrap [OS]  SNP stratified bootstrap [SS]  Double bootstrap [DB]  Jackknife [JK]  Robust HC1 [RB]  DM after MA [DM2] | 0.244  0.036  0.061  0.060  0.063  0.060  0.054  0.024  0.033 | 0.245  0.060  0.119  0.118  0.123  0.114  0.089  -0.210  0.066 | 0.246  0.178  0.432  0.439  0.436  0.399  0.245  -3.272  0.205 | 0.244  0.345  -1.581  -1.191  -1.727  -1.846  0.213  -10.835  0.399 |
| **Coverage**  Crude  DM before MA [DM1]  Basic bootstrap [BB]  Outcome stratified bootstrap [OS]  SNP stratified bootstrap [SS]  Double bootstrap [DB]  Jackknife [JK]  Robust HC1 [RB]  DM after MA [DM2] | 0.000  0.963  0.965  0.962  0.954  0.964  0.952  0.938  0.953 | 0.000  0.958  0.958  0.965  0.950  0.967  0.949  0.885  0.939 | 0.000  0.921  0.902  0.896  0.888  0.918  0.908  0.530  0.892 | 0.002  0.899  0.812  0.818  0.785  0.803  0.893  0.147  0.860 |
| **Mean SE**  Crude  DM before MA [DM1]  Basic bootstrap [BB]  Outcome stratified bootstrap [OS]  SNP stratified bootstrap [SS]  Double bootstrap [DB]  Jackknife [JK]  Robust HC1 [RB]  DM after MA [DM2] | 0.016  0.150  0.171  0.171  0.166  0.174  0.153  0.147  0.151 | 0.022  0.205  0.252  0.249  0.243  0.259  0.213  0.199  0.206 | 0.034  0.317  0.640  0.595  0.610  0.649  0.355  0.286  0.319 | 0.048  0.459  1.965  1.740  1.857  1.968  0.578  0.325  0.461 |
| **ESE**  Crude  DM before MA [DM1]  Basic bootstrap [BB]  Outcome stratified bootstrap [OS]  SNP stratified bootstrap [SS]  Double bootstrap [DB]  Jackknife [JK]  Robust HC1 [RB]  DM after MA [DM2] | 0.016  0.136  0.145  0.145  0.145  0.147  0.138  0.289  0.146 | 0.022  0.185  0.201  0.199  0.197  0.203  0.187  1.520  0.198 | 0.033  0.281  0.440  0.424  0.435  0.453  0.296  5.414  0.304 | 0.047  0.378  3.669  3.356  3.773  3.646  0.676  8.208  0.410 |
| **RMSE**  Crude  DM before MA [DM1]  Basic bootstrap [BB]  Outcome stratified bootstrap [OS]  SNP stratified bootstrap [SS]  Double bootstrap [DB]  Jackknife [JK]  Robust HC1 [RB]  DM after MA [DM2] | 0.244  0.141  0.158  0.157  0.158  0.158  0.148  0.290  0.150 | 0.246  0.194  0.233  0.231  0.233  0.232  0.207  1.535  0.208 | 0.248  0.333  0.617  0.611  0.616  0.603  0.385  6.326  0.366 | 0.249  0.512  3.995  3.561  4.150  4.086  0.709  13.593  0.572 |
| **Number of failed models**  Crude  DM before MA [DM1]  Basic bootstrap [BB]  Outcome stratified bootstrap [OS]  SNP stratified bootstrap [SS]  Double bootstrap [DB]  Jackknife [JK]  Robust HC1 [RB]  DM after MA [DM2] | 0  0  0  0  0  0  0  2  0 | 0  0  0  0  0  0  0  8  0 | 0  0  0  0  0  0  0  123  0 | 0  0  0  0  0  0  0  383  0 |
| **ESE – mean SE**  Crude  DM before MA [DM1]  Basic bootstrap [BB]  Outcome stratified bootstrap [OS]  SNP stratified bootstrap [SS]  Double bootstrap [DB]  Jackknife [JK]  Robust HC1 [RB]  DM after MA [DM2] | -0.001  -0.014  -0.026  -0.025  -0.021  -0.028  -0.014  0.142  -0.005 | 0.000  -0.020  -0.051  -0.050  -0.046  -0.056  -0.027  1.321  -0.008 | -0.001  -0.036  -0.200  -0.170  -0.175  -0.197  -0.059  5.128  -0.016 | -0.001  -0.080  1.704  1.616  1.916  1.678  0.099  7.883  -0.051 |

* MAF = minor allele frequency; DM = delta method; MA = meta-analysis; SNP = single nucleotide polymorphism ; SE = standard error; ESE = empirical standard error; RMSE = square root of the mean squared error. The crude model regresses the log(odds) of the dichotomous outcome on the continuous phenotype. The mean F-statistic for the IV-phenotype association is 24.66.

**Appendix table 4 Simulation results for scenario IV assessing performance of different instrumental variable variance estimators under different probabilities for the outcome with the MAF fixed at 0.01. ***

|  | Prob(y = 1) = 0.10 | Prob(y = 1) = 0.05 | Prob(y = 1) = 0.02 | Prob(y = 1) = 0.01 |
| --- | --- | --- | --- | --- |
| **Mean odds ratio (truth=1.000)**  Crude  DM before MA [DM1]  Basic bootstrap [BB]  Outcome stratified bootstrap [OS]  SNP stratified bootstrap [SS]  Double bootstrap [DB]  Jackknife [JK]  Robust HC1 [RB]  DM after MA [DM2] | 1.279  1.141  1.239  1.236  1.230  1.245  1.247  0.074  1.204 | 1.280  1.339  0.772  0.778  0.741  0.537  1.447  <0.001  1.572 | 1.284  2.181  0.013  0.014  0.011  0.008  0.171  <0.001  3.021 | 1.281  3.699  <0.001  <0.001  <0.001  <0.001  <0.001  <0.001  5.324 |
| **Mean bias**  Crude  DM before MA [DM1]  Basic bootstrap [BB]  Outcome stratified bootstrap [OS]  SNP stratified bootstrap [SS]  Double bootstrap [DB]  Jackknife [JK]  Robust HC1 [RB]  DM after MA [DM2] | 0.246  0.132  0.214  0.212  0.207  0.219  0.220  -2.605  0.186 | 0.247  0.292  -0.259  -0.251  -0.300  -0.622  0.370  -9.740  0.452 | 0.250  0.780  -4.324  -4.301  -4.502  -4.813  -1.765  -19.690  1.106 | 0.247  1.308  -9.525  -9.540  -10.020  -9.912  -9.437  -20.864  1.672 |
| **Coverage**  Crude  DM before MA [DM1]  Basic bootstrap [BB]  Outcome stratified bootstrap [OS]  SNP stratified bootstrap [SS]  Double bootstrap [DB]  Jackknife [JK]  Robust HC1 [RB]  DM after MA [DM2] | 0.000  0.968  0.984  0.985  0.967  0.986  0.943  0.524  0.929 | 0.000  0.942  0.961  0.965  0.936  0.951  0.922  0.165  0.859 | 0.000  0.867  0.795  0.809  0.732  0.744  0.793  0.003  0.690 | 0.004  0.793  0.573  0.572  0.486  0.493  0.386  0.000  0.550 |
| **Mean SE**  Crude  DM before MA [DM1]  Basic bootstrap [BB]  Outcome stratified bootstrap [OS]  SNP stratified bootstrap [SS]  Double bootstrap [DB]  Jackknife [JK]  Robust HC1 [RB]  DM after MA [DM2] | 0.017  0.326  1.397  1.397  1.000  1.358  0.368  0.282  0.346 | 0.022  0.456  3.769  3.736  2.712  3.384  0.599  0.288  0.483 | 0.034  0.905  7.178  7.026  5.678  5.464  1.476  0.214  0.776 | 0.048  8.753  8.861  8.997  7.035  6.171  2.516  0.201  8.670 |
| **ESE**  Crude  DM before MA [DM1]  Basic bootstrap [BB]  Outcome stratified bootstrap [OS]  SNP stratified bootstrap [SS]  Double bootstrap [DB]  Jackknife [JK]  Robust HC1 [RB]  DM after MA [DM2] | 0.017  0.260  0.578  0.645  0.636  0.844  0.294  4.862  0.324 | 0.023  0.345  2.312  2.282  2.370  4.171  0.619  7.871  0.442 | 0.035  0.515  6.154  5.888  6.072  5.500  4.938  6.122  0.604 | 0.050  2.994  7.351  7.380  7.316  6.673  8.682  4.493  3.266 |
| **RMSE**  Crude  DM before MA [DM1]  Basic bootstrap [BB]  Outcome stratified bootstrap [OS]  SNP stratified bootstrap [SS]  Double bootstrap [DB]  Jackknife [JK]  Robust HC1 [RB]  DM after MA [DM2] | 0.246  0.292  0.616  0.679  0.669  0.872  0.367  5.516  0.374 | 0.248  0.452  2.326  2.296  2.389  4.217  0.721  12.523  0.632 | 0.253  0.934  7.521  7.292  7.559  7.309  5.244  20.620  1.260 | 0.252  3.268  12.032  12.061  12.406  11.949  12.823  21.342  3.670 |
| **Number of failed models**  Crude  DM before MA [DM1]  Basic bootstrap [BB]  Outcome stratified bootstrap [OS]  SNP stratified bootstrap [SS]  Double bootstrap [DB]  Jackknife [JK]  Robust HC1 [RB]  DM after MA [DM2] | 0  0  0  0  0  0  0  93  0 | 0  0  0  0  0  0  0  365  0 | 0  0  0  0  0  0  0  782  0 | 0  0  0  0  0  0  0  1305  0 |
| **ESE – mean SE**  Crude  DM before MA [DM1]  Basic bootstrap [BB]  Outcome stratified bootstrap [OS]  SNP stratified bootstrap [SS]  Double bootstrap [DB]  Jackknife [JK]  Robust HC1 [RB]  DM after MA [DM2] | 0.000  -0.066  -0.820  -0.752  -0.363  -0.514  -0.074  4.580  -0.022 | 0.000  -0.111  -1.457  -1.454  -0.342  0.787  0.020  7.583  -0.041 | 0.000  -0.390  -1.024  -1.138  0.394  0.036  3.462  5.909  -0.171 | 0.002  -5.759  -1.510  -1.617  0.281  0.502  6.167  4.292  -5.404 |

* MAF = minor allele frequency; DM = delta method; MA = meta-analysis; SNP = single nucleotide polymorphism ; SE = standard error; ESE = empirical standard error; RMSE = square root of the mean squared error. The crude model regresses the log(odds) of the dichotomous outcome on the continuous phenotype. The mean F-statistic for the IV-phenotype association is 5.95.

**Appendix table 5 Sensitivity analysis repeating simulation scenario 1 with an increased mean sample size of 60,000 subjects. ***

|  | MAF = 0.500 | MAF = 0.100 | MAF = 0.050 | MAF = 0.010 | MAF = 0.005 |
| --- | --- | --- | --- | --- | --- |
| **Mean odds ratio (truth=1.000)**  Crude  DM before MA [DM1]  Basic bootstrap [BB]  Outcome stratified bootstrap [OS]  SNP stratified bootstrap [SS]  Double bootstrap [DB]  Jackknife [JK]  Robust HC1 [RB]  DM after MA [DM2] | 1.779  1.001  1.004  1.004  1.004  1.004  1.004  1.001  1.000 | 1.826  1.003  1.011  1.011  1.011  1.010  1.010  1.003  0.999 | 1.839  1.004  1.018  1.018  1.018  1.017  1.016  1.003  0.996 | 1.852  1.034  1.100  1.101  1.092  1.091  1.077  1.032  0.997 | 1.853  1.059  1.183  1.184  1.175  1.169  1.130  1.078  0.992 |
| **Mean bias**  Crude  DM before MA [DM1]  Basic bootstrap [BB]  Outcome stratified bootstrap [OS]  SNP stratified bootstrap [SS]  Double bootstrap [DB]  Jackknife [JK]  Robust HC1 [RB]  DM after MA [DM2] | 0.576  0.001  0.004  0.004  0.004  0.004  0.004  0.001  0.000 | 0.602  0.003  0.011  0.011  0.011  0.010  0.010  0.003  -0.001 | 0.609  0.004  0.017  0.017  0.018  0.017  0.016  0.003  -0.004 | 0.616  0.033  0.095  0.096  0.088  0.087  0.075  0.031  -0.003 | 0.617  0.057  0.168  0.169  0.161  0.157  0.122  0.075  -0.008 |
| **Coverage**  Crude  DM before MA [DM1]  Basic bootstrap [BB]  Outcome stratified bootstrap [OS]  SNP stratified bootstrap [SS]  Double bootstrap [DB]  Jackknife [JK]  Robust HC1 [RB]  DM after MA [DM2] | 0.000  0.958  0.954  0.952  0.954  0.954  0.953  0.957  0.956 | 0.000  0.955  0.945  0.946  0.947  0.947  0.943  0.952  0.952 | 0.000  0.952  0.938  0.938  0.937  0.939  0.929  0.946  0.941 | 0.000  0.966  0.922  0.921  0.887  0.935  0.918  0.944  0.956 | 0.000  0.971  0.929  0.933  0.909  0.940  0.899  0.910  0.962 |
| **ESE**  Crude  DM before MA [DM1]  Basic bootstrap [BB]  Outcome stratified bootstrap [OS]  SNP stratified bootstrap [SS]  Double bootstrap [DB]  Jackknife [JK]  Robust HC1 [RB]  DM after MA [DM2] | 0.007  0.023  0.023  0.023  0.023  0.023  0.023  0.023  0.023 | 0.007  0.039  0.040  0.040  0.039  0.040  0.039  0.039  0.039 | 0.007  0.054  0.055  0.055  0.055  0.055  0.054  0.053  0.054 | 0.007  0.118  0.142  0.142  0.126  0.141  0.117  0.115  0.119 | 0.007  0.166  0.269  0.270  0.240  0.252  0.165  0.159  0.170 |
| **Empirical SE**  Crude  DM before MA [DM1]  Basic bootstrap [BB]  Outcome stratified bootstrap [OS]  SNP stratified bootstrap [SS]  Double bootstrap [DB]  Jackknife [JK]  Robust HC1 [RB]  DM after MA [DM2] | 0.007  0.023  0.023  0.023  0.023  0.023  0.023  0.023  0.023 | 0.007  0.038  0.038  0.038  0.038  0.038  0.038  0.039  0.039 | 0.007  0.053  0.054  0.054  0.054  0.054  0.054  0.054  0.055 | 0.007  0.104  0.117  0.118  0.119  0.114  0.106  0.139  0.118 | 0.007  0.137  0.222  0.229  0.235  0.273  0.149  0.274  0.167 |
| **RMSE**  Crude  DM before MA [DM1]  Basic bootstrap [BB]  Outcome stratified bootstrap [OS]  SNP stratified bootstrap [SS]  Double bootstrap [DB]  Jackknife [JK]  Robust HC1 [RB]  DM after MA [DM2] | 0.576  0.023  0.023  0.023  0.023  0.023  0.023  0.023  0.023 | 0.602  0.038  0.040  0.040  0.040  0.040  0.039  0.039  0.039 | 0.609  0.053  0.057  0.057  0.057  0.057  0.056  0.054  0.055 | 0.616  0.109  0.151  0.153  0.148  0.144  0.130  0.143  0.118 | 0.617  0.149  0.278  0.284  0.285  0.315  0.193  0.284  0.167 |
| **Number of failed models**  Crude  DM before MA [DM1]  Basic bootstrap [BB]  Outcome stratified bootstrap [OS]  SNP stratified bootstrap [SS]  Double bootstrap [DB]  Jackknife [JK]  Robust HC1 [RB]  DM after MA [DM2] | 0  0  0  0  0  0  0  0  0 | 0  0  0  0  0  0  0  0  0 | 0  0  0  0  0  1  0  0  0 | 0  0  0  0  0  0  0  1  0 | 0  4  4  4  4  4  4  7  4 |
| **ESE – mean SE**  Crude  DM before MA [DM1]  Basic bootstrap [BB]  Outcome stratified bootstrap [OS]  SNP stratified bootstrap [SS]  Double bootstrap [DB]  Jackknife [JK]  Robust HC1 [RB]  DM after MA [DM2] | 0.000  -0.001  -0.001  -0.001  0.000  -0.001  0.000  0.000  0.000 | 0.000  -0.001  -0.001  -0.001  -0.001  -0.001  -0.001  0.000  0.000 | 0.000  -0.001  -0.001  -0.001  -0.001  -0.001  0.000  0.001  0.001 | 0.000  -0.014  -0.025  -0.024  -0.007  -0.026  -0.011  0.024  -0.001 | 0.000  -0.028  -0.047  -0.041  -0.004  0.021  -0.016  0.116  -0.003 |

* MAF = minor allele frequency; DM = delta method; MA = meta-analysis; SNP = single nucleotide polymorphism ; SE = standard error; ESE = empirical standard error; RMSE = square root of the mean squared error. The crude model regresses the log(odds) of the dichotomous outcome on the continuous phenotype. The mean F-statistics for the IV-phenotype association are 375.98, 136.00, 72.20, 15.98, and 8.55.

**Appendix table 6 Sensitivity analysis repeating simulation scenario 1 with a mean sample size of 20,000 subjects using a one stage meta-analysis design. ***

|  | MAF = 0.500 | MAF = 0.100 | MAF = 0.050 | MAF = 0.010 | MAF = 0.005 |
| --- | --- | --- | --- | --- | --- |
| **Mean odds ratio (truth=1.000)**  Crude  Delta method [DM]  Basic bootstrap [BB]  Outcome stratified bootstrap [OS]  SNP stratified bootstrap [SS]  Double bootstrap [DB]  Jackknife [JK]  Robust HC1 [RB]  Percentile Method | 1.780  1.000  1.000  1.000  1.000  1.000  1.000  1.000  1.000 | 1.827  1.000  1.000  1.000  1.000  1.000  1.000  1.000  1.000 | 1.840  0.997  0.997  0.997  0.997  0.997  0.997  0.997  0.997 | 1.853  0.991  0.991  0.991  0.991  0.991  0.991  0.991  0.991 | 1.855  0.984  0.984  0.984  0.984  0.984  0.984  0.984  0.984 |
| **Mean bias**  Crude  Delta method [DM]  Basic bootstrap [BB]  Outcome stratified bootstrap [OS]  SNP stratified bootstrap [SS]  Double bootstrap [DB]  Jackknife [JK]  Robust HC1 [RB]  Percentile Method | 0.576  0.000  0.000  0.000  0.000  0.000  0.000  0.000  0.000 | 0.603  0.000  0.000  0.000  0.000  0.000  0.000  0.000  0.000 | 0.610  -0.003  -0.003  -0.003  -0.003  -0.003  -0.003  -0.003  -0.003 | 0.617  -0.009  -0.009  -0.009  -0.009  -0.009  -0.009  -0.009  -0.009 | 0.618  -0.016  -0.016  -0.016  -0.016  -0.016  -0.016  -0.016  -0.016 |
| **Coverage**  Crude  Delta method [DM]  Basic bootstrap [BB]  Outcome stratified bootstrap [OS]  SNP stratified bootstrap [SS]  Double bootstrap [DB]  Jackknife [JK]  Robust HC1 [RB]  Percentile Method | 0.000  0.955  0.957  0.955  0.956  0.955  0.956  0.954  0.955 | 0.000  0.955  0.955  0.953  0.950  0.954  0.953  0.954  0.954 | 0.000  0.951  0.953  0.954  0.899  0.954  0.953  0.950  0.947 | 0.000  0.958  0.962  0.963  0.868  0.960  0.951  0.944  0.943 | 0.000  0.975  0.977  0.978  0.952  0.977  0.968  0.955  0.958 |
| **Mean SE**  Crude  Delta method [DM]  Basic bootstrap [BB]  Outcome stratified bootstrap [OS]  SNP stratified bootstrap [SS]  Double bootstrap [DB]  Jackknife [JK]  Robust HC1 [RB]  Percentile Method | 0.012  0.041  0.041  0.041  0.041  0.041  0.041  0.041  NA | 0.012  0.068  0.068  0.068  0.067  0.068  0.068  0.068  NA | 0.012  0.093  0.094  0.094  0.080  0.094  0.093  0.093  NA | 0.013  0.211  0.227  0.227  0.174  0.224  0.213  0.209  NA | 0.013  0.307  13.775  0.891  0.859  0.436  0.310  0.298  NA |
| **ESE**  Crude  Delta method [DM]  Basic bootstrap [BB]  Outcome stratified bootstrap [OS]  SNP stratified bootstrap [SS]  Double bootstrap [DB]  Jackknife [JK]  Robust HC1 [RB]  Percentile Method | 0.012  0.040  0.040  0.040  0.040  0.040  0.040  0.040  0.040 | 0.013  0.067  0.067  0.067  0.067  0.067  0.067  0.067  0.067 | 0.013  0.094  0.094  0.094  0.094  0.094  0.094  0.094  0.094 | 0.013  0.218  0.218  0.218  0.218  0.218  0.218  0.218  0.218 | 0.013  0.312  0.312  0.312  0.312  0.312  0.312  0.312  0.312 |
| **RMSE**  Crude  Delta method [DM]  Basic bootstrap [BB]  Outcome stratified bootstrap [OS]  SNP stratified bootstrap [SS]  Double bootstrap [DB]  Jackknife [JK]  Robust HC1 [RB]  Percentile Method | 0.577  0.040  0.040  0.040  0.040  0.040  0.040  0.040  0.040 | 0.603  0.067  0.067  0.067  0.067  0.067  0.067  0.067  0.067 | 0.610  0.094  0.094  0.094  0.094  0.094  0.094  0.094  0.094 | 0.617  0.218  0.218  0.218  0.218  0.218  0.218  0.218  0.218 | 0.618  0.313  0.313  0.313  0.313  0.313  0.313  0.313  0.313 |
| **Number of failed models**  Crude  Delta method [DM]  Basic bootstrap [BB]  Outcome stratified bootstrap [OS]  SNP stratified bootstrap [SS]  Double bootstrap [DB]  Jackknife [JK]  Robust HC1 [RB]  Percentile Method | 0  0  0  0  0  0  0  0  0 | 0  0  0  0  0  0  0  0  0 | 0  0  0  0  0  0  0  0  0 | 0  0  0  0  0  0  0  0  0 | 0  0  0  0  0  0  0  0  0 |
| **ESE – mean SE**  Crude  Delta method [DM]  Basic bootstrap [BB]  Outcome stratified bootstrap [OS]  SNP stratified bootstrap [SS]  Double bootstrap [DB]  Jackknife [JK]  Robust HC1 [RB]  Percentile Method | 0.000  -0.001  -0.001  -0.001  -0.001  -0.001  -0.001  -0.001  -0.001  -0.001 | 0.000  0.000  -0.001  -0.001  0.000  -0.001  0.000  0.000  0.000  -0.001 | 0.000  0.001  0.000  0.000  0.014  0.000  0.001  0.001  0.001  0.000 | 0.000  0.007  -0.009  -0.009  0.044  -0.006  0.005  0.009  0.007  -0.009 | 0.000  0.006  -13.463  -0.578  -0.547  -0.124  0.002  0.014  0.006  -0.061 |

# The basic bootstrap percentile method does not estimate a standard error. * MAF = minor allele frequency; DM = delta method; MA = meta-analysis; SNP = single nucleotide polymorphism ; SE = standard error; ESE = empirical standard error; RMSE = square root of the mean squared error. The crude model regresses the log(odds) of the dichotomous outcome on the continuous phenotype. The mean F-statistics for the IV-phenotype association 125.33, 45.95, 24.84, 5.93, and 3.55.

**Appendix table 7 Sensitivity analysis repeating simulation scenario 1 with between study variance, a mean sample size of 20,000 subjects, and using a two stage meta-analysis design. ***

|  | MAF = 0.500 | MAF = 0.100 | MAF = 0.050 | MAF = 0.010# | MAF = 0.005 |
| --- | --- | --- | --- | --- | --- |
| **Mean odds ratio (truth=1.000)**  Crude  DM before MA [DM1]  Basic bootstrap [BB]  Outcome stratified bootstrap [OS]  SNP stratified bootstrap [SS]  Double bootstrap [DB]  Jackknife [JK]  Robust HC1 [RB]  DM after MA [DM2] | 1.375  1.001  1.003  1.002  0.905  1.025  1.004  1.002  0.978 | 1.418  1.002  1.006  1.006  1.006  1.016  1.007  1.002  0.996 | 1.437  1.006  1.012  1.012  1.011  1.005  1.014  1.006  0.996 | 1.453  1.019  1.007  1.035  1.023  1.044  1.042  1.021  15.21*10^9^ | 1.454  1.033  1.050  1.140  1.069  1.020  1.066  1.041  0.773 |
| **Mean bias**  Crude  DM before MA [DM1]  Basic bootstrap [BB]  Outcome stratified bootstrap [OS]  SNP stratified bootstrap [SS]  Double bootstrap [DB]  Jackknife [JK]  Robust HC1 [RB]  DM after MA [DM2] | 0.318  0.001  0.003  0.002  -0.100  0.025  0.004  0.002  -0.023 | 0.349  0.002  0.006  0.006  0.006  0.016  0.006  0.002  -0.004 | 0.363  0.006  0.012  0.012  0.011  0.005  0.013  0.006  -0.004 | 0.374  0.019  0.007  0.035  0.022  0.043  0.041  0.021  23.445 | 0.374  0.033  0.049  0.131  0.067  0.019  0.064  0.040  -0.257 |
| **Coverage**  Crude  DM before MA [DM1]  Basic bootstrap [BB]  Outcome stratified bootstrap [OS]  SNP stratified bootstrap [SS]  Double bootstrap [DB]  Jackknife [JK]  Robust HC1 [RB]  DM after MA [DM2] | 0.000  0.966  0.962  0.963  0.960  0.962  0.964  0.971  0.999 | 0.000  0.955  0.951  0.950  0.949  0.949  0.950  0.958  0.996 | 0.000  0.963  0.955  0.957  0.953  0.960  0.957  0.962  0.997 | 0.000  0.973  0.971  0.971  0.957  0.973  0.957  0.964  0.999 | 0.000  0.981  0.983  0.985  0.981  0.984  0.964  0.955  0.998 |
| **Mean SE**  Crude  DM before MA [DM1]  Basic bootstrap [BB]  Outcome stratified bootstrap [OS]  SNP stratified bootstrap [SS]  Double bootstrap [DB]  Jackknife [JK]  Robust HC1 [RB]  DM after MA [DM2] | 0.031  0.022  0.022  0.023  0.024  0.024  0.022  0.023  24.865 | 0.032  0.036  0.036  0.035  0.036  0.037  0.036  0.038  5.416 | 0.032  0.049  0.050  0.049  0.049  0.051  0.049  0.054  17.452 | 0.034  0.102  0.148  0.152  0.132  0.158  0.106  0.124  19.60*10^5^ | 0.034  0.142  0.426  0.481  0.336  0.474  0.172  0.281  43.80*10 |
| **ESE**  Crude  DM before MA [DM1]  Basic bootstrap [BB]  Outcome stratified bootstrap [OS]  SNP stratified bootstrap [SS]  Double bootstrap [DB]  Jackknife [JK]  Robust HC1 [RB]  DM after MA [DM2] | 0.037  0.020  0.021  0.054  4.683  1.341  0.020  0.021  1.681 | 0.037  0.034  0.038  0.035  0.037  0.459  0.035  0.036  0.972 | 0.038  0.047  0.048  0.048  0.078  0.278  0.048  0.051  1.338 | 0.039  0.094  1.347  0.146  0.503  0.810  0.111  0.236  10.51*10^2^ | 0.040  0.123  3.026  5.466  1.780  5.307  0.224  0.890  10.286 |
| **RMSE**  Crude  DM before MA [DM1]  Basic bootstrap [BB]  Outcome stratified bootstrap [OS]  SNP stratified bootstrap [SS]  Double bootstrap [DB]  Jackknife [JK]  Robust HC1 [RB]  DM after MA [DM2] | 0.320  0.020  0.021  0.054  4.684  1.341  0.021  0.021  1.681 | 0.351  0.034  0.038  0.035  0.038  0.459  0.035  0.036  0.972 | 0.365  0.047  0.050  0.049  0.079  0.278  0.050  0.051  1.338 | 0.376  0.096  1.347  0.150  0.503  0.811  0.118  0.237  10.51*10^10^ | 0.377  0.127  3.027  5.467  1.782  5.307  0.233  0.891  10.289 |
| **Number of failed models**  Crude  DM before MA [DM1]  Basic bootstrap [BB]  Outcome stratified bootstrap [OS]  SNP stratified bootstrap [SS]  Double bootstrap [DB]  Jackknife [JK]  Robust HC1 [RB]  DM after MA [DM2] | 0  0  0  0  0  0  0  0  0 | 0  0  0  0  0  0  0  0  0 | 0  0  0  0  0  0  0  0  0 | 0  0  0  0  0  0  0  2  0 | 0  5  5  5  5  5  5  28  5 |
| **ESE – mean SE**  Crude  DM before MA [DM1]  Basic bootstrap [BB]  Outcome stratified bootstrap [OS]  SNP stratified bootstrap [SS]  Double bootstrap [DB]  Jackknife [JK]  Robust HC1 [RB]  DM after MA [DM2] | 0.005  -0.002  -0.001  0.031  4.659  1.316  -0.002  -0.002  -23.184 | 0.006  -0.001  0.002  -0.001  0.001  0.422  -0.001  -0.002  -4.444 | 0.006  -0.002  -0.002  -0.002  0.029  0.227  -0.001  -0.003  -16.113 | 0.005  -0.008  1.199  -0.006  0.371  0.652  0.005  0.112  -195.978*10^4^ | 0.007  -0.019  2.600  4.985  1.444  4.833  0.052  0.609  -427.721 |

# The large deviation of the DM2 method seen at a MAF of 0.010 is due to an single estimated log odds ratio of 46988.78, excluding this value results in a mean OR, mean bias, and empirical SE of 0.952, -0.049, 29.869 respectively. * MAF = minor allele frequency; DM = delta method; MA = meta-analysis; SNP = single nucleotide polymorphism ; SE = standard error; ESE = empirical standard error; RMSE = square root of the mean squared error. The crude model regresses the log(odds) of the dichotomous outcome on the continuous phenotype. The mean F-statistics for the IV-phenotype association are 408.31, 147.15, 77.54, 17.16, 9.02.

**Appendix table 8 Sensitivity analysis repeating simulation scenario 1 with additional variance estimators. ***

|  | MAF = 0.500 | MAF = 0.100 | MAF = 0.050 | MAF = 0.010# | MAF = 0.005 |
| --- | --- | --- | --- | --- | --- |
| **Mean odds ratio (truth=1.000)**  Crude  TJ before MA [DM2]  TJ after MA [TJ2]  DM with BB before MA [DM1 BB]  DM with BB after MA [DM2 BB] | 1.778  1.003  0.999  1.002  0.999 | 1.825  1.010  0.998  1.009  0.998 | 1.839  1.018  0.998  1.013  0.998 | 1.850  1.066  0.978  1.106  0.976 | 1.854  1.126  0.966  1.238  1.177 |
| **Mean bias**  Crude  TJ before MA [DM2]  TJ after MA [TJ2]  DM with BB before MA [DM1 BB]  DM with BB after MA [DM2 BB] | 0.576  0.003  -0.001  0.002  -0.001 | 0.602  0.010  -0.002  0.009  -0.002 | 0.609  0.018  -0.002  0.013  -0.002 | 0.615  0.064  -0.022  0.101  -0.024 | 0.617  0.119  -0.035  0.213  0.163 |
| **Coverage**  Crude  TJ before MA [DM2]  TJ after MA [TJ2]  DM with BB before MA [DM1 BB]  DM with BB after MA [DM2 BB] | 0.000  0.950  0.949  0.954  0.947 | 0.000  0.960  0.957  0.966  0.961 | 0.000  0.949  0.949  0.962  0.953 | 0.000  0.942  0.946  0.969  0.952 | 0.000  0.940  0.967  0.889  0.859 |
| **Mean SE**  Crude  TJ before MA [DM2]  TJ after MA [TJ2]  DM with BB before MA [DM1 BB]  DM with BB after MA [DM2 BB] | 0.012  0.040  0.041  0.041  0.041 | 0.012  0.067  0.067  0.105  0.068 | 0.012  0.091  0.093  0.151  0.094 | 0.013  0.192  0.210  238.758  0.228 | 0.013  0.261  0.313  28.277  0.757 |
| **ESE**  Crude  TJ before MA [DM2]  TJ after MA [TJ2]  DM with BB before MA [DM1 BB]  DM with BB after MA [DM2 BB] | 0.012  0.040  0.040  0.040  0.040 | 0.013  0.065  0.067  0.085  0.067 | 0.013  0.092  0.094  0.108  0.094 | 0.012  0.190  0.215  3.274  0.297 | 0.013  0.243  0.311  2.183  4.730 |
| **RMSE**  Crude  TJ before MA [DM2]  TJ after MA [TJ2]  DM with BB before MA [DM1 BB]  DM with BB after MA [DM2 BB] | 0.576  0.040  0.040  0.041  0.040 | 0.602  0.066  0.067  0.086  0.067 | 0.610  0.093  0.094  0.108  0.094 | 0.616  0.201  0.216  3.275  0.298 | 0.617  0.270  0.313  2.193  4.733 |
| **Number of failed models**  Crude  TJ before MA [DM2]  TJ after MA [TJ2]  DM with BB before MA [DM1 BB]  DM with BB after MA [DM2 BB] | 0  0  0  0  0 | 0  0  0  0  0 | 0  0  0  0  0 | 0  0  0  0  0 | 0  22  22  22  22 |
| **ESE – mean SE**  Crude  TJ before MA [DM2]  TJ after MA [TJ2]  DM with BB before MA [DM1 BB]  DM with BB after MA [DM2 BB] | 0.000  0.000  0.000  -0.001  0.000 | 0.000  -0.001  -0.001  -0.020  -0.001 | 0.000  0.000  0.000  -0.043  0.000 | 0.000  -0.002  0.005  -235.485  0.069 | 0.000  -0.018  -0.001  -26.094  3.973 |

* MAF = minor allele frequency; TJ = Toby Johnson; MA = meta-analysis; BB = basic bootstrap; SE = standard error; ESE = empirical standard error; RMSE = square root of the mean squared error. The crude model regresses the log(odds) of the dichotomous outcome on the continuous phenotype. The mean F-statistics for the IV-phenotype association are 125.45, 46.14, 24.76, 5.99, 3.45.

Appendix table 9 Sensitivity analysis repeating simulation scenario 1 with using a continuous outcome. *

|  | MAF = 0.500 | MAF = 0.100 | MAF = 0.050 | MAF = 0.010 | MAF = 0.005 |
| --- | --- | --- | --- | --- | --- |
| **Mean, mean difference (truth=0.000)**  Crude  DM before MA [DM1]  Basic bootstrap [BB]  SNP stratified bootstrap [SS]  Double bootstrap [DB]  Jackknife [JK]  Robust HC1 [RB]  DM after MA [DM2] | 0.706  0.005  0.016  0.016  0.016  0.015  0.005  -0.001 | 0.741  0.031  0.090  0.091  0.086  0.074  0.027  -0.002 | 0.749  0.191  0.386  0.382  0.341  0.342  0.223  -0.033 | 0.748  0.113  0.277  0.265  0.266  0.224  0.108  -0.021 | 0.749  0.191  0.386  0.382  0.341  0.342  0.223  -0.033 |
| **Mean bias**  Crude  DM before MA [DM1]  Basic bootstrap [BB]  SNP stratified bootstrap [SS]  Double bootstrap [DB]  Jackknife [JK]  Robust HC1 [RB]  DM after MA [DM2] | 0.706  0.005  0.016  0.016  0.016  0.015  0.005  -0.001 | 0.734  0.014  0.046  0.046  0.044  0.040  0.013  -0.003 | 0.741  0.031  0.090  0.091  0.086  0.074  0.027  -0.002 | 0.748  0.113  0.277  0.265  0.266  0.224  0.108  -0.021 | 0.749  0.191  0.386  0.382  0.341  0.342  0.223  -0.033 |
| **Coverage**  Crude  DM before MA [DM1]  Basic bootstrap [BB]  SNP stratified bootstrap [SS]  Double bootstrap [DB]  Jackknife [JK]  Robust HC1 [RB]  DM after MA [DM2] | 0.000  0.949  0.921  0.923  0.922  0.921  0.946  0.952 | 0.000  0.953  0.885  0.878  0.885  0.884  0.944  0.947 | 0.000  0.947  0.831  0.816  0.836  0.836  0.932  0.956 | 0.000  0.943  0.820  0.778  0.807  0.684  0.873  0.958 | 0.000  0.923  0.834  0.776  0.759  0.602  0.728  0.974 |
| **Mean SE**  Crude  DM before MA [DM1]  Basic bootstrap [BB]  SNP stratified bootstrap [SS]  Double bootstrap [DB]  Jackknife [JK]  Robust HC1 [RB]  DM after MA [DM2] | 0.007  0.036  0.037  0.037  0.037  0.036  0.036  0.036 | 0.007  0.061  0.063  0.063  0.063  0.060  0.060  0.061 | 0.007  0.083  0.091  0.089  0.090  0.081  0.081  0.084 | 0.007  0.181  0.392  0.325  0.306  0.164  0.164  0.190 | 0.007  0.246  0.934  0.743  0.530  0.212  0.200  0.281 |
| **ESE**  Crude  DM before MA [DM1]  Basic bootstrap [BB]  SNP stratified bootstrap [SS]  Double bootstrap [DB]  Jackknife [JK]  Robust HC1 [RB]  DM after MA [DM2] | 0.007  0.036  0.037  0.037  0.037  0.037  0.037  0.037 | 0.007  0.058  0.060  0.060  0.060  0.059  0.060  0.061 | 0.007  0.075  0.082  0.082  0.081  0.079  0.080  0.083 | 0.007  0.140  0.220  0.219  0.199  0.151  0.167  0.194 | 0.007  0.180  0.420  0.496  2.100  0.240  0.322  0.300 |
| **RMSE**  Crude  DM before MA [DM1]  Basic bootstrap [BB]  SNP stratified bootstrap [SS]  Double bootstrap [DB]  Jackknife [JK]  Robust HC1 [RB]  DM after MA [DM2] | 0.706  0.037  0.040  0.040  0.040  0.040  0.037  0.037 | 0.734  0.060  0.075  0.075  0.074  0.071  0.061  0.061 | 0.741  0.081  0.122  0.123  0.118  0.108  0.085  0.083 | 0.748  0.180  0.354  0.344  0.333  0.270  0.199  0.195 | 0.749  0.263  0.570  0.627  2.127  0.418  0.392  0.302 |
| **Number of failed models**  Crude  DM before MA [DM1]  Basic bootstrap [BB]  SNP stratified bootstrap [SS]  Double bootstrap [DB]  Jackknife [JK]  Robust HC1 [RB]  DM after MA [DM2] | 0  0  0  0  0  0  0  0 | 0  0  0  0  0  0  0  0 | 0  0  0  0  0  0  0  0 | 0  0  0  0  0  0  0  0 | 0  12  12  12  12  12  12  12 |
| **ESE – mean SE**  Crude  DM before MA [DM1]  Basic bootstrap [BB]  SNP stratified bootstrap [SS]  Double bootstrap [DB]  Jackknife [JK]  Robust HC1 [RB]  DM after MA [DM2] | 0.000  0.000  0.000  0.000  0.000  0.001  0.001  0.001 | 0.000  -0.003  -0.004  -0.003  -0.003  -0.001  0.000  0.001 | 0.000  -0.008  -0.009  -0.006  -0.009  -0.002  -0.001  0.000 | 0.000  -0.041  -0.172  -0.106  -0.107  -0.014  0.004  0.004 | 0.000  -0.066  -0.514  -0.246  1.570  0.027  0.121  0.019 |

* MAF = minor allele frequency; DM = delta method; MA = meta-analysis; SNP = single nucleotide polymorphism ; SE = standard error; ESE = empirical standard error; RMSE = square root of the mean squared error. The crude model regresses the log(odds) of the dichotomous outcome on the continuous phenotype. The mean F-statistics for the IV-phenotype association are: 125.63, 46.04, 24.93, 5.94, 3.49

**Appendix table 10 Baseline characteristics of a 6 study IPDMA using SNPs rs11591147 and rs2965101 in an instrumental variables analysis of the LDL-C effect on CVD*.**

|  | **BRHS** | | **CaPS** | | **EAS** | | **ELSA** | |
| --- | --- | --- | --- | --- | --- | --- | --- | --- |
|  | Mean(sd) | n | Mean(sd) | n | Mean(sd) | n | Mean(sd) | n |
| **CVD** | 0.34 | 802 | 0.17 | 182 | 0.67 | 510 | 0.86 | 1624 |
| **Men** | 1.00 | 2342 | 1.00 | 1087 | 0.48 | 370 | 0.53 | 993 |
| **Age** (years) | 68.91(5.62) | 2342 | 56.77(4.46) | 1065 | 64.51(5.64) | 764 | 73.69(9.44) | 1883 |
| **Systolic blood pressure** (mm Hg) | 144.17(19.95) | 2340 | 145.78(22.40) | 1061 | 143.42(23.76) | 763 | 139.01(19.66) | 1662 |
| **Diastolic blood pressure** (mm Hg) | 81.88(12.86) | 2340 | 84.56(12.01) | 1061 | 77.46(12.20) | 761 | 72.97(11.43) | 1662 |
| **Cholesterol** (mmol/L) | 6.37(1.04) | 2331 | 5.63(1.00) | 1031 | 7.11(1.34) | 763 | 5.71(1.28) | 1873 |
| **HDL-C** (mmol/L) | 1.15(0.25) | 2245 | 1.03(0.25) | 1031 | 1.45(0.37) | 760 | 1.49(0.39) | 1872 |
| **LDL-C** (mmol/L) | 3.89(1.00) | 2277 | 3.75(0.90) | 1006 | 5.35(1.24) | 760 | 3.43(1.06) | 1835 |
| **Triglycerides** (mmol/L) | 2.06(1.23) | 1500 | 1.92(1.14) | 1031 | 1.53(0.87) | 763 | 1.80(1.11) | 1873 |
| **rs11591147** (n rare alleles)  0  1  2 | 0.97  0.03  0.00 | 2261  81  0 | 0.97  0.03  0.00 | 1050  37  0 | 0.97  0.11  0.00 | 739  81  0 | 0.97  0.06  0.00 | 1819  121  0 |
| **rs2965101** (n rare alleles)  0  1  2 | 0.46  0.44  0.10 | 1073  1023  245 | 0.46  0.44  0.10 | 498  477  112 | 0.46  0.44  0.03 | 351  334  25 | 0.48  0.42  0.03 | 906  795  64 |
| **Total sample size** |  | 2342 |  | 1087 |  | 764 |  | 1883 |

Appendix table 10 continued.

|  | **MRC46** |  | **WHII** |  | **Total** |  |
| --- | --- | --- | --- | --- | --- | --- |
|  | Mean(sd) | n | Mean(sd) | n | Mean(sd) | n |
| **CVD** | 0.06 | 144 | 0.14 | 409 | 0.18 | 2624 |
| **Men** | 0.50 | 1231 | 0.23 | 713 | 0.56 | 8422 |
| **Age** (years) | 53.00(0.00) | 2464 | 48.94(5.98) | 3041 | 59.63(11.21) | 11559 |
| **Systolic blood pressure** (mm Hg) | 136.22(20.01) | 2425 | 120.37(13.01) | 3034 | 135.40(21.20) | 11285 |
| **Diastolic blood pressure** (mm Hg) | 84.55(12.17) | 2425 | 79.63(9.11) | 3034 | 80.49(12.08) | 11283 |
| **Cholesterol** (mmol/L) | 6.09(1.07) | 2314 | 6.44(1.13) | 3040 | 6.21(1.20) | 11352 |
| **HDL-C** (mmol/L) | 1.67(0.52) | 2149 | 1.41(0.40) | 3023 | 1.39(0.44) | 11087 |
| **LDL-C** (mmol/L) | 3.52(0.97) | 2139 | 4.37(1.01) | 2980 | 3.96(1.14) | 11004 |
| **Triglycerides** (mmol/L) | 2.16(1.51) | 2310 | 1.44(1.15) | 3041 | 1.80(1.26) | 10517 |
| **rs11591147** (n rare alleles)  0  1  2 | 0.96  0.04  0.00 | 2368  94  2 | 0.97  0.03  0.00 | 2947  92  2 | 0.97  0.03  0.00 | 11184  393  4 |
| **rs11206510** (n rare alleles)  0  1  2 | 0.47  0.44  0.10 | 1154  1074  236 | 0.45  0.45  0.10 | 1362  1373  306 | 0.46  0.44  0.10 | 5327  5093  1160 |
| **Total sample size** |  | 2464 |  | 3041 |  | 11581 |

* The baseline numbers are based on complete data on CVD, and SNPS.

Appendix figure 1 Flowchart of the implementation of the different variance estimators in a simulation study of a two-stage meta-analysis of an instrumental variable analysis*.


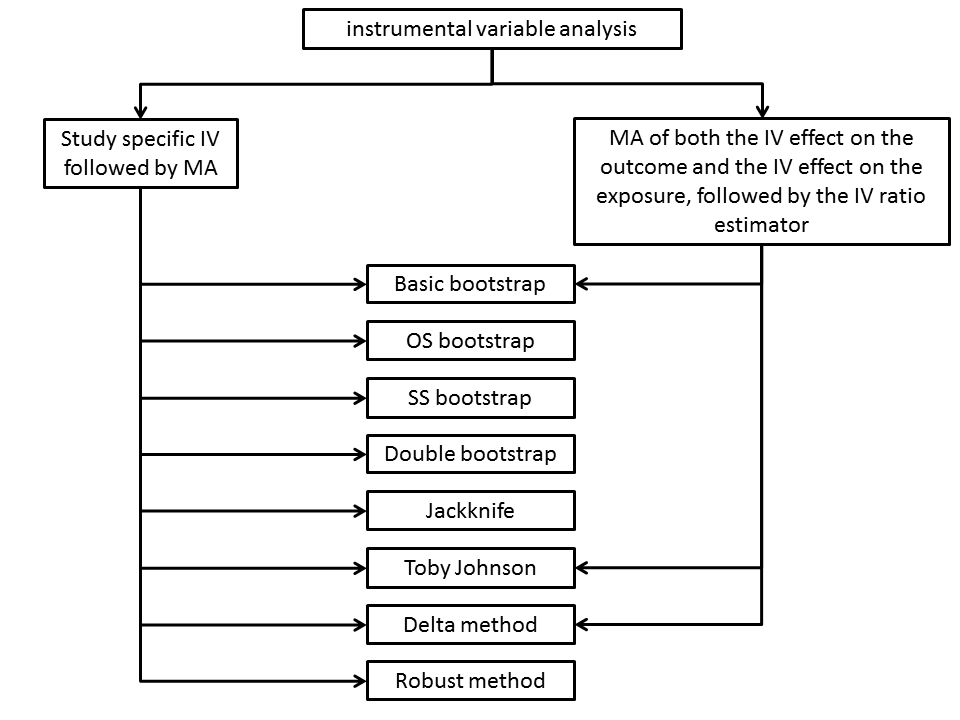


*MA; meta-analysis, SS; SNP stratified, OS; outcome stratified.

**Appendix Figure 2 Spearman pairwise correlation matrix for PCSK9 SNPs rs13465, rs6511720, and multiple phenotypes; with p-values for non-significant associations depicted (alpha = 0.05).**

**
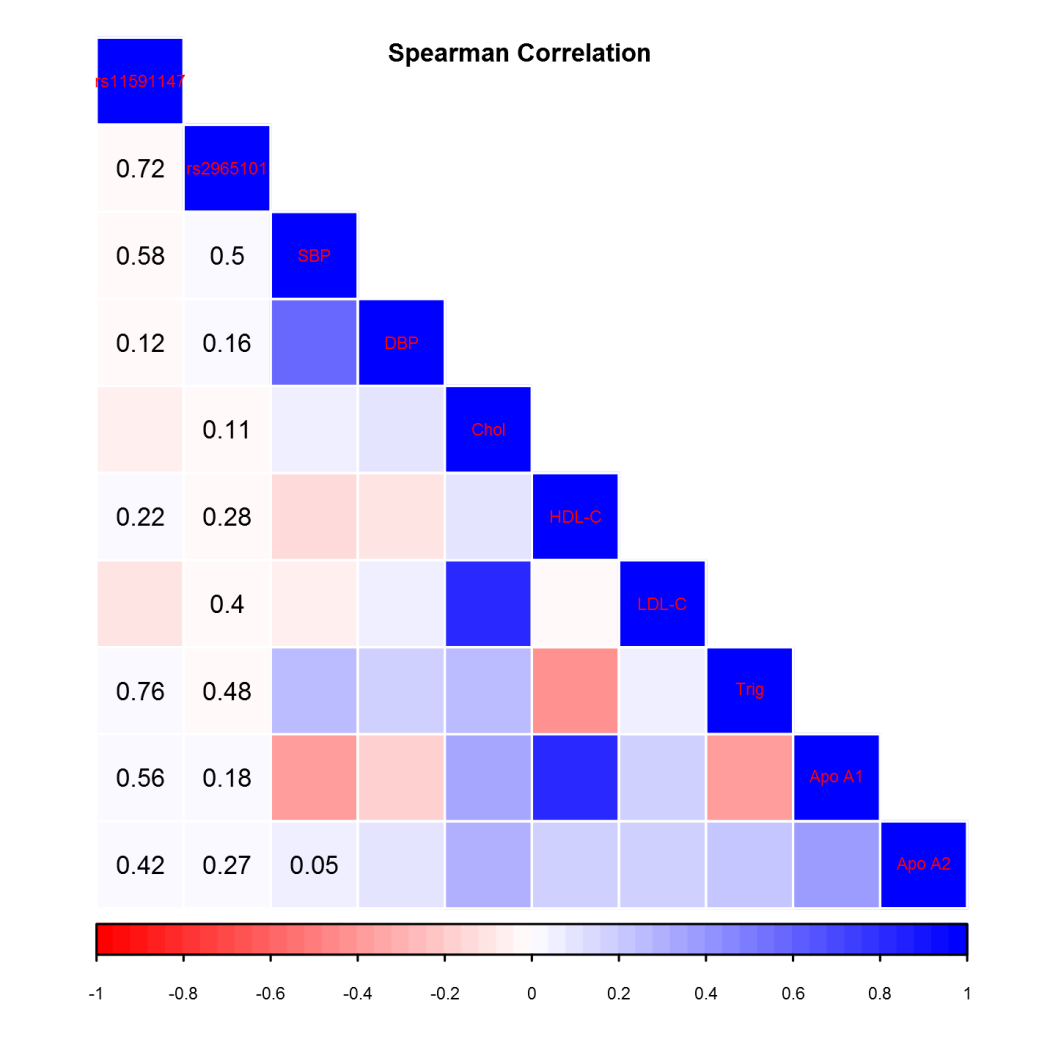
**

Reference List

(1) Shah T, Engmann J, Dale C, Shah S, White J, Giambartolomei C, et al. Population genomics of cardiometabolic traits: design of the University College London-London School of Hygiene and Tropical Medicine-Edinburgh-Bristol (UCLEB) Consortium. PLoS One 2013;8(8):e71345.

(2) Shaper AG, Pocock SJ, Walker M, Cohen NM, Wale CJ, Thomson AG. British Regional Heart Study: cardiovascular risk factors in middle-aged men in 24 towns. Br Med J (Clin Res Ed) 1981 Jul 18;283(6285):179-86.

(3) Bainton D, Miller NE, Bolton CH, Yarnell JWG, Sweetnam PM, Baker IA, et al. Plasma triglyceride and high density lipoprotein cholesterol as predictors of ischaemic heart disease in British men: The Caerphilly and Speedwell Collaborative Heart Disease Studies. Br Heart J 1992 Jul;68(1):60-6.

(4) Fowkes FG, Housley E, Cawood EH, Macintyre CC, Ruckley CV, Prescott RJ. Edinburgh Artery Study: prevalence of asymptomatic and symptomatic peripheral arterial disease in the general population. Int J Epidemiol 1991 Jun;20(2):384-92.

(5) Marmot MG, Banks J, Blundell R, Lessof C, Nazroo J. Health, wealth and lifestyles of the older population in England: ELSA 2002. 2003.

(6) Kuh D, Pierce M, Adams J, Deanfield J, Ekelund U, Friberg P, et al. Cohort profile: updating the cohort profile for the MRC National Survey of Health and Development: a new clinic-based data collection for ageing research. Int J Epidemiol 2011 Feb;40(1):e1-e9.

(7) Marmot MG, Davey Smith G, Stansfeld S, Patel C, North F, Head J, et al. Health inequalities among British civil servants: the Whitehall II study. Lancet 1991 Jun 8;337(8754):1387-93.

(8) Schmidt AF, Groenwold RH, Knol MJ, Hoes AW, Nielen M, Roes KC, et al. Exploring interaction effects in small samples increases rates of false-positive and false-negative findings: results from a systematic review and simulation study. J Clin Epidemiol 2014 Jul;67(7):821-9.

(9) Higgins JP, Thompson SG. Quantifying heterogeneity in a meta-analysis. Stat Med 2002 Jun 15;21(11):1539-58.
