# Supplementary figures and images for: Comparison of variance estimators for meta-analysis of instrumental variable estimates
Source: Int J Epidemiol. 2016 Sep 2;45(6):1975–86. doi: 10.1093/ije/dyw123 (PMC5654757; doi:10.1093/ije/dyw123)

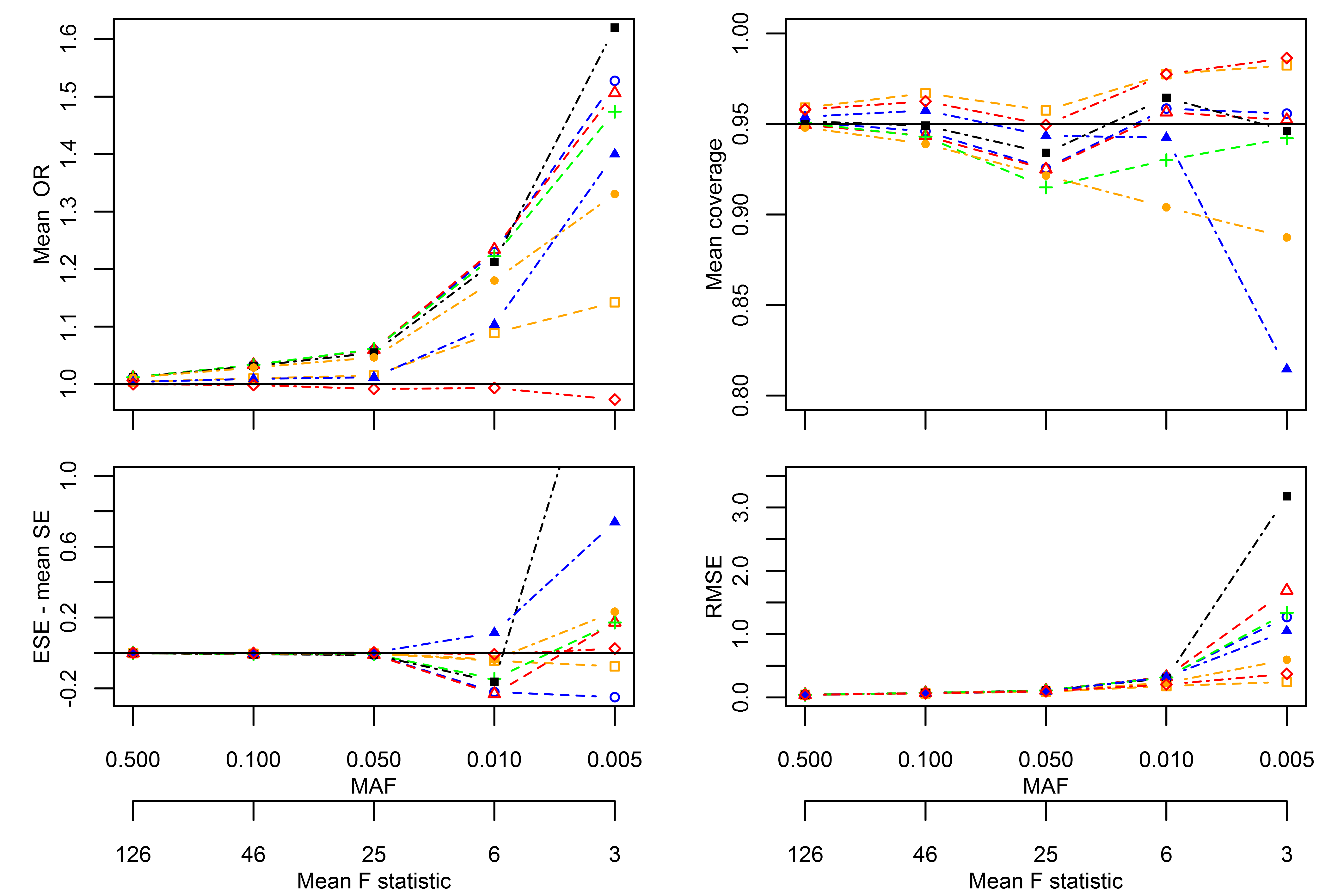

Supplement: Supplementary Data [file dyw123_supplementary_data.zip › ije-2015-09-1226-File008.tif]

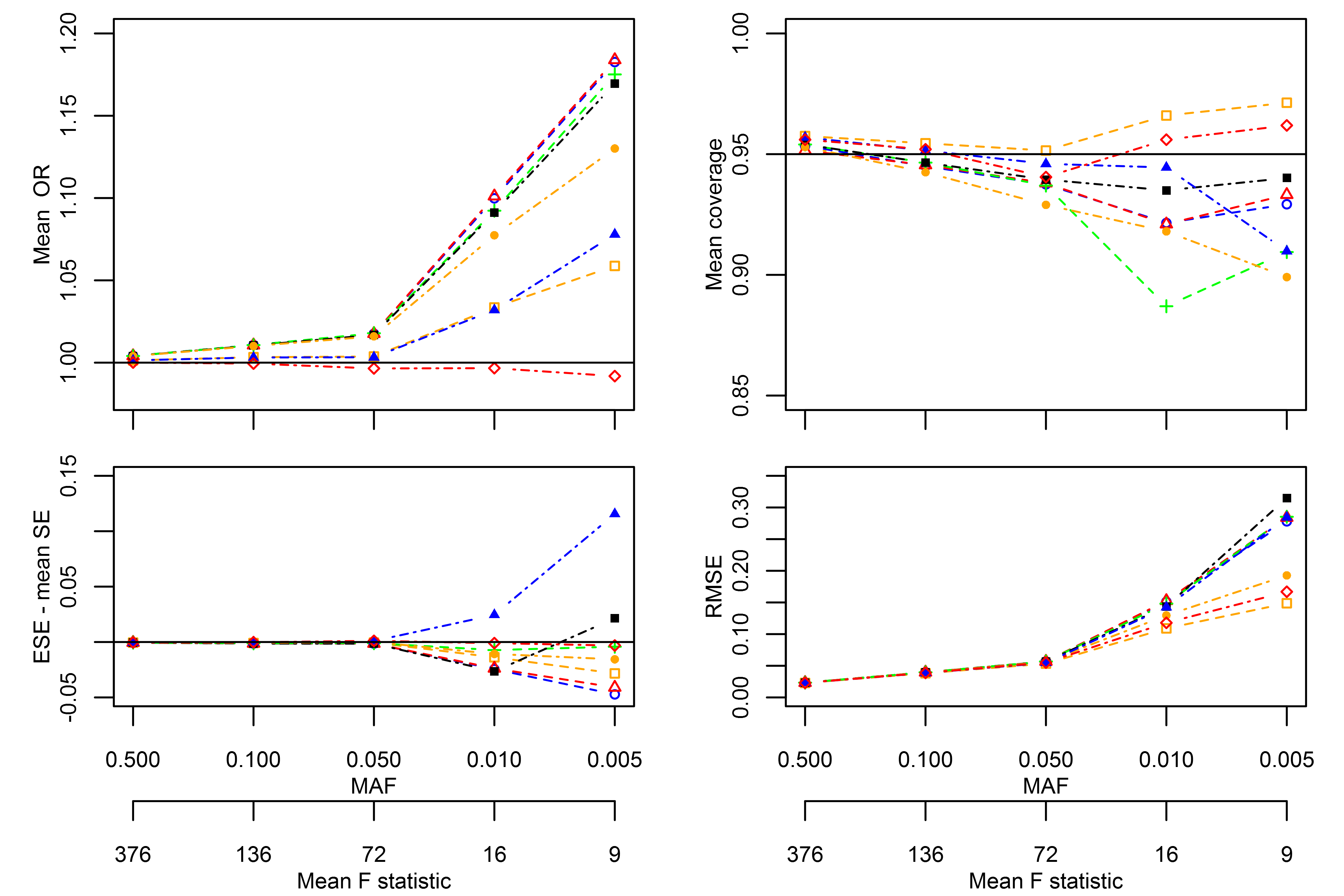

Supplement: Supplementary Data [file dyw123_supplementary_data.zip › ije-2015-09-1226-File009.tif]

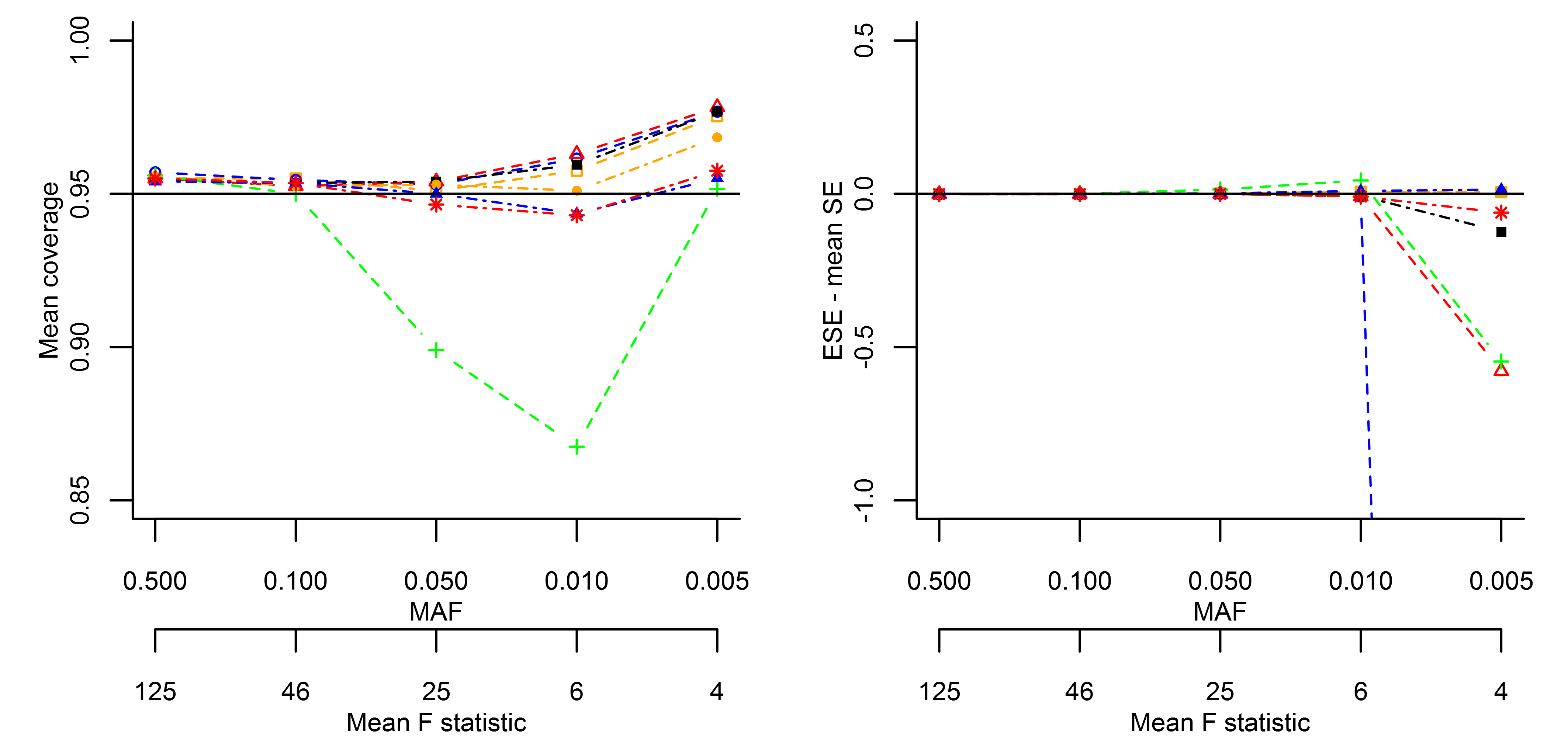

Supplement: Supplementary Data [file dyw123_supplementary_data.zip › ije-2015-09-1226-File010.tif]
